# Transgene Expression in Microalgae—From Tools to Applications

**DOI:** 10.3389/fpls.2016.00505

**Published:** 2016-04-22

**Authors:** Lior Doron, Na'ama Segal, Michal Shapira

**Affiliations:** Department of Life Sciences, Ben-Gurion University of the NegevBeer Sheva, Israel

**Keywords:** *Chlamydomonas*, chloroplast transformation of algae, diatoms, microalgae, nuclear transformation of algae, red microalgae, selection markers

## Abstract

Microalgae comprise a biodiverse group of photosynthetic organisms that reside in water sources and sediments. The green microalgae *Chlamydomonas reinhardtii* was adopted as a useful model organism for studying various physiological systems. Its ability to grow under both photosynthetic and heterotrophic conditions allows efficient growth of non-photosynthetic mutants, making *Chlamydomonas* a useful genetic tool to study photosynthesis. In addition, this green alga can grow as haploid or diploid cells, similar to yeast, providing a powerful genetic system. As a result, easy and efficient transformation systems have been developed for *Chlamydomonas*, targeting both the chloroplast and nuclear genomes. Since microalgae comprise a rich repertoire of species that offer variable advantages for biotech and biomed industries, gene transfer technologies were further developed for many microalgae to allow for the expression of foreign proteins of interest. Expressing foreign genes in the chloroplast enables the targeting of foreign DNA to specific sites by homologous recombination. Chloroplast transformation also allows for the introduction of genes encoding several enzymes from a complex pathway, possibly as an operon. Expressing foreign proteins in the chloroplast can also be achieved by introducing the target gene into the nuclear genome, with the protein product bearing a targeting signal that directs import of the transgene-product into the chloroplast, like other endogenous chloroplast proteins. Integration of foreign genes into the nuclear genome is mostly random, resulting in large variability between different clones, such that extensive screening is required. The use of different selection modalities is also described, with special emphasis on the use of herbicides and metabolic markers which are considered to be friendly to the environment, as compared to drug-resistance genes that are commonly used. Finally, despite the development of a wide range of transformation tools and approaches, expression of foreign genes in microalgae suffers from low efficiency. Thus, novel tools have appeared in recent years to deal with this problem. Finally, while *C. reinhardtii* was traditionally used as a model organism for the development of transformation systems and their subsequent improvement, similar technologies can be adapted for other microalgae that may have higher biotechnological value.

## Introduction

Microalgae first attracted the attention of the biotech industry several decades ago as a potential platform for extracting natural products, with further improvements in their production relying on advanced technologies. Microalgae provide a manufacturing platform for the expression of foreign genes due to their quick growth under photoautotrophic conditions and their low cost of maintenance, as compared to land plants, mammalian cells, yeast, and bacteria. In addition, many algal species have developed unique metabolic pathways that produce compounds of commercial value (Priyadarshani and Rath, 2012; Rasala and Mayfield, 2014). Certain algal species, such as *Dunaliella*, are adapted for growth under extreme conditions, in this case high salinity (Ben-Amotz and Avron, 1972), thereby reducing the risk of contamination.

The plethora of high-value chemicals that can be extracted from both cyanobacteria and eukaryote microalgae include compounds used in the food industry, medicine, and cosmetics. The possibility of advancing the use of microalgae for future development of biofuel production and additional health-related products has also been examined. Microalgae have been exploited for the production of food additives, such as β-carotene (Varela et al., 2015) and astaxanthine, as well as long-chain polyunsaturated fatty acids (PUFAs; Borowitzka, 2013; Sharon-Gojman et al., 2015). Another line of industrial exploitation makes use of cell-wall sulfated polysaccharides, mainly for the cosmetics industry (Arad and Levy-Ontman, 2010). However, competition from cheaper parallel compounds extracted from macroalgae reveals the need for improvements to microlalgal systems. Efforts have long been directed at the industrial use of microalgal biomass for biofuel production, although the relatively high cost and low yield are of concern. Thus, biotechnological improvements combined with global market changes may, however, have major implications for the future use of microalgae as a biofuel source. Algae can also contribute in the development of novel and sensitive biosensors for environmental uses, such as monitoring pollutants in soil and water sources (Viji et al., 2014; Diaz et al., 2015). Finally, algae are an important component of the marine aquaculture, and can be used in ecological reef rehabilitation, especially in view of their ability to form symbiontic relationships with coral (Hagedorn and Carter, 2015).

Heterotrophic growth of microalgae is usually limited to bioreactors, whereas photoautotrophic growth can be carried out in open ponds. Still, the use of open ponds is restricted to extreme conditions (salt, temperature, pH) that allow exclusive growth of the organism of interest and avoid contamination by opportunistic organisms. This has been shown for *Dunaliella*, which is adopted to grow at high salt concentrations (Ben-Amotz and Avron, 1983), as well as for *Spirulina*, a photoautotrophic cyanobacteria that grows at high pH (Nolla-Ardevol et al., 2015).

In view of the above, genetic manipulations of microalga can lead to major changes in microalgal biotechnology-based industries. Such manipulations include the transfer of biosynthetic cascades into organisms that are suitable for growth in open ponds, or alternatively, adapting organisms that produce compounds of interest to grow under extreme conditions, thus reducing the risk of contamination. Recent developments in genome sequencing, combined with old and new systems for genetic manipulation, act synergistically to advance all aspects of microalgal research, both basic and applied. This review focuses on available systems and approaches for genetic manipulation of the chloroplast and nuclear genomes of microalgae, addressing unsolved issues, as well as points that await improvement.

Significant efforts have been invested in establishing tools that will allow for realization of the promise that algae hold for the production of high value bio-products, with most such tools having been originally developed for the model alga *Chlamydomonas reinhardtii* (Kindle et al., 1989). Transgene expression in *Chlamydomonas* was based on the accumulated identification of regulatory elements, such as promoters and untranslated regions (UTRs; Harris, 2009). Subsequently, successful nuclear transformation systems were also developed for ~25 microalgae species (see Tables 1, 2). In many cases, microalgal transformation resulted in stable expression of transgenes from either the nuclear or plastid genomes. The large amount of genomic and EST data from different algae contribute to the rich molecular toolbox currently available.

**Table 1:** **List of selection markers and selection modes for chloroplast transformation**.

**Type of selection marker**	**Selection gene**	**Gene product**	**Selection mode**	**Species/Genetic background**	**References**
Antibiotic resistance	*aadA*	Aminoglycoside 3′ adenylyltransferase	Resistance to Spectinomycin/Streptomycin	*Chlamydomonas reinhardtii*	Goldschmidt-Clermont, 1991
				*Euglena gracilis*	Doetsch et al., 2001
				*Haematococcus pluvialis*	Gutiérrez et al., 2012
	*aphA6*	Aminoglycoside 3′- transferase	Resistance to Kanamycin	*Chlamydomonas reinhardtii*	Bateman and Purton, 2000
	*ereB*	Erythromycin esterase	Resistance to Erythromycin	*Dunaliella tertiolecta*	Georgianna et al., 2013
	*rrnS* and *rrnL* point mutation	16S and 23S	Resistance to Spectinomycin, Streptomycin, Kanamycin and Erythromycin	*Chlamydomonas reinhardtii*	Newman et al., 1990
	*Cat*	Chloramphenicol acetyltransferase	Resistance to Chloramphenicol	*Phaeodactylum tricornutum*	Xie et al., 2014
Herbicide resistance	*psbA* mutant	Photosystem II protein D1	Resistance to 3-(3,4-Dichlorophenyl)-1,1-dimethylurea (DCMU)	*Chlamydomonas reinhardtii*	Przibilla et al., 1991; Newman et al., 1992
	*ahas*(W492S)	Acetohydroxyacid synthase	Resistance to Sulfometuron methyl	*Porphyridium* sp.	Lapidot et al., 1999, 2002
				*Parietochloris incisa*	Grundman et al., 2012
	*Bar*	Phosphinothricin acetyltransferase	Tolerance to Glufosinate, or its ammonium salt DL- Phosphinothricin	*Platymonas subcordiformis*	Cui et al., 2014
Metabolic markers	*atpB*	β subunit of ATP synthase	Photoautotrophic growth	*Chlamydomonas reinhardtii* cc-373 (*atpB-*)	Boynton et al., 1988
	*nifH*	β-glucuronidase	Photoautotrophic growth	*Chlamydomonas reinhardtii* (*chlL*-)	Cheng et al., 2005
	*psbA*	Photosystem II protein D1	Photoautotrophic growth	*Chlamydomonas reinhardtii* FUD7	Michelet et al., 2011
	*tscA*	Small RNA that participates in trans-splicing of the *psaA* transcript	Photoautotrophic growth	*Chlamydomonas reinhardtii* (*tscA*-)	Goldschmidt-Clermont, 1991; Kindle et al., 1991
	*arg9*	Acetylornithine aminotransferase	Arginine free media	*Chlamydomonas reinhardtii* (*arg9-*)	Remacle et al., 2009

**Table 2:** **Commonly used nuclear control elements for constitutive or inducible transgene expression**.

**Functional elements**	**Originally controlling expression of**	**Property**	**Source of element**	**Comments**	**Algal species used with this control element**
CaMV35S promoter	35S Viral protein from the Cauliflower mosaic virus	Strong heterologous constitutive promoter which functions well in land plants and some algae species	Cauliflower mosaic virus	Contradictory data for *Chlamydomonas*	*Chlamydomonas reinhardtii*, Successful transformation: (Kumar et al., 2004)
					Unsuccessful transformation: (Day et al., 1990; Diaz-Santos et al., 2013)
				High efficiency for different *Chlorella* species, *Dunalliela*, diatoms, Haematoccoccus and *Nanochloropsis*	*Chlorella kessleri*, (El-Sheekh, 1999)
					*Chlorella ellipsoidea*, (Jarvis and Brown, 1991)
					*Chlorella vulgaris*, (Chow and Tung, 1999; Wang C. et al., 2007; Cha et al., 2012)
					*Dunaliella salina* (Tan et al., 2005)
					*Amphidinium* sp. *and Symbiodinium microadriaticum*, (ten Lohuis and Miller, 1998)
					*Phaeodactylum tricornutum, (Sakaue et al., 2008)*
					*Haematococcus pluvialis*, (Kathiresan et al., 2015)
					*Nannochloropsis* sp., (Cha et al., 2011)
RBCS2 promoter	Small subunit of the ribulose bisphosphate carboxylase	Strong endogenous constitutive promoter	*Chlamydomonas reinhardtii*	Introducing the first intron of *RBCS2* into the coding region or fusing the HSP70A promoter upstream to the RBCS2 promoter greatly improves the expression of transgenes	*Chlamydomonas reinhardtii*, (Stevens et al., 1996; Lumbreras et al., 1998; Fuhrmann et al., 1999; Schroda et al., 2000)
					*Dunaliella salina*, (Sun et al., 2005)
					*Chlorella ellipsoidea*, (Kim et al., 2002)
					*Volvox carteri*, (Hallmann and Wodniok, 2006)
					*Pseudochoricystis ellipsoidea*, (Imamura et al., 2012)
					*Nannochloropsis* sp., (Chen et al., 2008; Li and Tsai, 2009)
			*Dunaliella salina*	Adding a nuclear matrix attachment regions (MAR) before the promoter region and after the terminator sequence was shown to increase transgene expression	*Dunaliella salina* (Wang T. Y. et al., 2007)
			*Dunaliella tertiolecta*	Only one transgenic line was recovered when used to transform *Dunaliella tertiolecta*	*Chlamydomonas reinhardtii*, (Walker et al., 2005a)
					*Dunaliella tertiolecta*, (Walker et al., 2005b)
			*Lobosphaera (Parietochloris) incisa*	The upstream region of the *Lobosphaera incisa* RBCS promoter (ranging from −1000 to −450) contains elements counteracting transformation or gene expression	*Chlamydomonas reinhardtii*, (Zorin et al., 2014)
					*Lobosphaera (Parietochloris) incise*, (Zorin et al., 2014)
*PSAD* promoter	An abundant chloroplast protein of Photosystem I complex, encoded by the nuclear genome	A strong endogenous constitutive promoter	*Chlamydomonas reinhardtii*	Expression driven by the *PSAD* promoter can be enhanced by high light	*Chlamydomonas reinhardtii*, (Fischer and Rochaix, 2001)
*FCP* promoter	Fucoxanthin-chlorophyll binding protein of the light-harvesting antennae complexes	Strong Endogenous and constitutive promoter	*Phaeodactylum tricornutum*	*FCP* promoters (A-E) are capable of driving expression of the bacterial *ble* gene at levels sufficient to confer resistance to Zeocin	*Phaeodactylum tricornutum*, (Apt et al., 1996; Falciatore et al., 1999; Zaslavskaia et al., 2000; Kilian and Kroth, 2005)
					*Thalassiosira weissflogii*, (Falciatore et al., 1999)
			*Cylindrotheca fusiformis*		*Cylindrotheca fusiformis*, (Poulsen and Kroger, 2005)
				*Thalassiosira pseudonana*	*Thalassiosira pseudonana*, (Poulsen et al., 2006)
				The transformation of *Cylindrotheca fusiformis* using a p*FCP*-based vector improved the transformation efficiency about four fold as compared to the Pδ-containing vectors	
Pδ	ε Frustulin–member of the calcium-binding glycoproteins	Endogenous constitutive promoter	*Cylindrotheca fusiformis*	Used to drive functional expression of a membrane protein	*Cylindrotheca fusiformis*, (Fischer et al., 1999)
*Ubi1*-Ω promoter	Ubiquitin promoter fused with the TMV-omega translation enhancer element	Strong heterologous constitutive promoter	*Zea maize*	Highly efficient for transformation of *Chlorella ellipsoidea* and *Dunaliella salina* cells	*Chlorella ellipsoidea*, (Chen et al., 2001)
					*Dunaliella salina*, (Geng et al., 2003, 2004)
GAPDH	Glyceraldehyde -3-phosphate dehydrogenase	Endogenous constitutive promoter	*Dunaliella salina*	Used to drive expression of the heterologous gene encoding bialaphos resistance (*bar*) and of the *N*-terminal fragment of human canstatin	*Dunaliella salina*, (Jia et al., 2012)
*CABII-1*	Light-harvesting chlorophyll a/b-binding proteins of photosystem II	Endogenous promoter	*Chlamydomonas reinhardtii*	The *NIT1* gene expressed under the control of the *CABII-1* promoter was highly stimulated by light	*Chlamydomonas reinhardtii*, (Blankenship and Kindle, 1992)
*NIT1*	Nitrate reductase promoter	Strong inducible endogenous promoter	*Chlamydomonas reinhardt*	Expression of the nitrate reductase is switched off when cells are grown in the presence of ammonium ions and becomes switched on within 4 h when cells are transferred to a medium containing nitrate. An expression vector with the *NIT1* promoter is widely used in many studies	*Chlamydomonas reinhardtii*, (Ohresser et al., 1997; Koblenz and Lechtreck, 2005; Schmollinger et al., 2010)
			*Chlorella ellipsoidea*		*Chlorella ellipsoidea*, (Wang et al., 2004)
			*Phaeodactylum tricornutum*		*Phaeodactylum tricornutum*, (Niu et al., 2012); *Chlorella vulgaris*, (Niu et al., 2011)
			*Dunaliella salina*		*Dunaliella salina*, (Li et al., 2007, 2008)
			*Cylindrotheca fusiformis*		*Cylindrotheca fusiformis*, (Poulsen and Kroger, 2005); *Phaeodactylum tricornutum*, (Miyagawa et al., 2009)
			*Thalassiosira pseudonana*		*Thalassiosira pseudonana*, (Poulsen et al., 2006)
			*Volvox carteri*		*Volvox carteri*, (von der Heyde et al., 2015)
LIP	Light-induced protein		*Dunaliella* sp.	The LIP promoter can be used for conditional gene expression in response to high light	*Chlamydomonas reinhardtii*, (Park et al., 2013; Baek et al., 2016)
B12-responsive element	B12-independent methionine synthase (METE)		*Chlamydomonas reinhardtii*	The B12-responsive element can repress expression of a reporter gene following the addition of B_12_	*Chlamydomonas reinhardtii*, (Helliwell et al., 2014)
TPP riboswitch	Thiamine pyrophosphate (TPP)	A riboswitch-regulated element based on the thiamine pyrophosphate	*Chlamydomonas reinhardtii*	The TPP riboswitch regulates expression in response to the presence or absence of TPP in the growth medium	*Chlamydomonas reinhardtii*, (Ramundo et al., 2013)
CYC6 promoter	Cytochrome c6	An inducible endogenous promoter		Metal-responsive element that is responsive to both nickel and cobalt ions and could be inhibited by EDTA	*Chlamydomonas reinhardtii*, (Quinn and Merchant, 1995)

## Chloroplast transformation systems

### Technical approaches used for chloroplast transformation

Although stable transformation of microalgae was first developed for the chloroplast of *C. reinhardtii*, there are still fewer transformation systems that target the chloroplast, as compared to those targeting the microalgal nucleus. One great advantage of chloroplast transformation is that transgenes can be easily directed to integrate via homologous recombination, whereas nuclear transformation of microalgae usually results in random integration events. The development of the CRISPR-CAS9 system in microalgae may offer a solution for targeting transgenes into specific sites in the nuclear genome. The improvement of chloroplast transformation tools is also a dynamic field, as discussed elsewhere for microalgae (Purton, 2007; Purton et al., 2013) and higher plants (Bock, 2015).

The first stable transformation system for the chloroplast of *C. reinhardtii* was established using biolistic delivery. The foreign DNA was designed to rescue three mutants of the chloroplast *atpB* gene by homologous recombination of the transgenic marker into the target mutant strain and restore photosynthetic activity (Boynton et al., 1988). It was further shown that *Chlamydomonas* chloroplast transformation could be achieved by agitating cell wall-deficient cells with the DNA of interest in the presence of glass beads (Kindle et al., 1991; Economou et al., 2014; Rochaix et al., 2014). A chloroplast transformation system that was based on integration into the inverted repeat of the plastid genome using electroporation was also developed for *Phaeodactylum tricornutum* (Xie et al., 2014). Thus, similar technologies can be used for both chloroplast and nuclear transformations. The various markers for chloroplast transformation systems available today are summarized in Table 1.

### Selection systems

#### Selection markers for chloroplast transformation based on photoautotrophic growth

*Chlamydomonas* offers the advantage of being able to grow under non-photosynthetic conditions, using acetate as a carbon energy source. Thus, using non-photosynthetic mutants as recipient strains and recovery of their photosynthetic activity as a reporter system was used for reconstituting expression of the mutated *atpB* gene, encoding for ATP synthase (Boynton et al., 1988), and the *tscA* gene, encoding a small RNA that participates in trans-splicing of the *psaA* transcript (Goldschmidt-Clermont, 1991; Kindle et al., 1991).

Marker rotation is an approach that was originally aimed at examining whether the bacterial *nifH* gene from *Klebsiella pneumoniae* could replace the algal *ChlL* gene, which is responsible for chlorophyll biosynthesis in the dark. Both genes show a remarkable similarity in their domain structure, suggesting that *nifH* could replace ChlL for binding to a [4Fe–4S] cluster, thereby directly introducing the nitrogenase Fe protein into the *Chlamydomonas* plastome. In addition to using this approach for investigating the nitrogenase-like complex in the chloroplast, it could serve as a platform for plastid engineering into a functional nitrogenase-containing organelle. Accordingly, *petB* (cytochrome b6) was initially replaced by the selection marker *aadA*, and non-photosynthetic transformants were selected. A second round of transformation with the mutant strain restored the *petB* gene product and, moreover, introduced *nifH* (or *uidA*, encoding β-glucuronidase), allowing selection based on the ability to grow under photoautotrophic conditions (Cheng et al., 2005).

The use of FUD7 as a recipient strain offered a selection mechanism based on the ability of the transformed algae to grow under photoautotrophic conditions. FUD7 is a mutant that carries a deletion between exon 1 of *psbA* and the 5S gene. Thus, a cassette that reinstalls the open reading frame of *psbA*, along with a tagged gene of interest flanked by 3′UTR sequences, was established (Michelet et al., 2011).

#### Selection markers for chloroplast transformation based on metabolic enzymes

Metabolic selection of transformed cells provides a great advantage over the use of genes encoding for resistance to antibiotics and herbicides, as metabolic selection is considered to be more environmental friendly. Metabolic selection is common in yeast and animal cells, especially since many markers offer positive and negative selections. For instance, acetylornithine aminotransferase (*ARG9*) is a key enzyme in the metabolic pathway of arginine. It is encoded in the nucleus of *C. reinhardtii*, with the protein translocating into the chloroplast. A mutation in *ARG9* resulted in an auxotrophic phenotype, such that the algal cells could only grow if arginine was added to the medium. The *Arabidopsis thaliana ARG9* gene, known for its high A/T content that is typical of the chloroplast genome (Nakamura et al., 2000), was expressed in the plastid of a *Chlamydomonas* mutant strain that was originally deficient of *ARG9* expression. The foreign *ARG9* gene, now encoded by the chloroplast genome, was able to rescue the auxotrophic phenotype and restore arginine synthesis. This elegant approach created a metabolic selection system for chloroplast transformation in *Chlamydomonas* (Remacle et al., 2009).

#### Selection markers for chloroplast transformation based on resistance to antibiotics

Mutations in the sequence of the 16S (*rrnS*) and 23S (*rrnL*) rRNA that confer resistance to spectinomycin, streptomycin, kanamycin and erythromycin in *C. reinhardtii* (Harris et al., 1989) were previously used to establish an antibiotic-based selective marker that exchanged wild type RNA with the gene encoding resistant RNA (Newman et al., 1990). Later, resistance to spectinomycin was conferred by introducing the bacterial-derived *aadA* gene into the chloroplast genome, encoding aminoglycoside 3′ adenyl transferase. This gene is still the most frequently used marker for chloroplast transformations in algae and higher plants. The *aadA-rbcL* expression cassette was adapted and used to transform the genome of the *Haematococcus pluvialis* chloroplast (Gutiérrez et al., 2012).

An additional bacterial gene, *aphA6*, encoding aminoglycoside (3′) transferase that confers resistance to aminoglycoside antibiotics was used with *Chlamydomnas reinhardtii*. The combination of *aadA* with *aphA6* extended the possibility for expressing multiple foreign genes in the chloroplast (Bateman and Purton, 2000). In 2013, the group of Stephan Mayfield constructed a cassette for *Dunaliella tertiolecta* chloroplast transformation. The construct that encodes both the gene of interest and the erythromycin esterase gene, *ereB*, was successfully introduced into *D. tertiolecta* cells via particle bombardment (Georgianna et al., 2013). A chloroplast-based expression system developed for the diatom *P. tricornutum* uses the chloramphenicol acetyltransferase (CAT) gene as a selection marker, as it confers resistance to chloramphenicol. This cassette promoted the expression of a foreign gene, along with the selection marker (Xie et al., 2014).

Finally, there is a strong demand for marker removal systems due to biotechnological concerns and commercial requests. Such a system was first established for *Chlamydomonas* by designing a dedicated cassette for promoting a recombination event that allows for removal of the selection marker, once the selection pressure is removed. The additional foreign gene in this cassette was maintained in the genome despite the removal of the selection gene marker because it was located outside of the sequence repeats that drive the recombination event (Fischer et al., 1996).

#### Selection markers for chloroplast transformation based on resistance to herbicides

A mutation in the fifth exon of the *psbA* coding region conferred resistance of *Chlamydomonas* cells to 3-(3,4-dichlorophenyl)-1,1-dimethylurea (DCMU), a herbicide that blocks the electron transfer pathway in PSII. Thus, DCMU resistance was initially used as a selection marker for understanding the occurrence of integration hotspots in the chloroplast genome (Newman et al., 1992). A different mutation in *psbA* also provides the cells with herbicide resistance to metribuzin (Przibilla et al., 1991).

The herbicide sulfometuron methyl (SMM) that inhibits growth of bacteria, yeast, algae and plants is commonly used as a selection marker in plants and algae. The target of SMM is the gene encoding acetohydroxyacid synthase (AHAS), an enzyme involved in the biosynthesis of branched amino acids. Since AHAS is encoded by the chloroplast genome of the red microalgae *Porphyridium* sp., it was found to be an ideal marker for chloroplast transformation. As such, a naturally occurring W492S mutation in the algal AHAS gene that confers resistance to SMM was used to establish a transformation system for *Porphyridium* sp. (Lapidot et al., 1999, 2002). During evolution, the gene encoding AHAS moved to the nucleus in land plants and green algae (Mazur et al., 1987), therefore allowing for its use as a selection marker in nuclear transformations in plants (Li et al., 1992; Ott et al., 1996) and green algae (Kovar et al., 2002). The AHAS-encoding gene was also later used as a transformation marker for the green microalgae *Lobosphaera (Parietochloris) incisa*, an algae with added value for biotechnological purposes (Grundman et al., 2012). In this case, the endogenous gene was first cloned, the W605S mutation in the active site of the enzyme that confers resistance to SMM was introduced, and the mutated gene was used for nuclear transformation.

Another chloroplast transformation system based on resistance to herbicides was developed for *Platymonas subcordiformis* using the bacterial *bar* gene encoding phosphinothricin acetyltransferase, which confers tolerance to glufosinate, or its ammonium salt, DL-phosphinothricin. This latter compound is the active ingredient in several herbicides, including the widely used Basta. The *bar* gene was also described as a selection marker in tobacco (Lutz et al., 2001). The use of this system in algae resulted in the development of a useful tool and a suitable selection system for this algae that is not sensitive to most commonly used antibiotics, such as spectinomycin, streptomycin or kanamycin (Cui et al., 2014).

Finally, as will be discussed below (see Section Biotechnological Exploitation of Microalgae), antibiotic and herbicide markers may raise biosafety concerns when genetic engineering is recruited for biotechnological purposes. Thus, selections involving metabolic markers that offer functional complementation of a missing endogenous gene product provide a clear advantage.

### Regulatory elements: promoters, UTRs, and codon optimization

Efficient expression of foreign genes in algal chloroplasts is usually best obtained through the use of endogenous regulatory elements, derived from genes that are abundantly expressed. Thus, promoters that drive expression of the large subunit of ribulose bisphosphate carboxylase/oxygenase (*rbcL)*, the D1 protein of the photosystem II reaction center (*psbA)*, and the Y subunit of ATP synthase (*atpA)*, were recruited for this purpose. The highest expression of soluble GUS was recorded for the *atpA* promoter and the 5′UTR (Ishikura et al., 1999). In another study, the *atpA* and *psbD* promoters and 5′UTRs were shown to drive the highest expression of a GFP reporter gene, when compared to the promoter and 5′UTRs of the *rbcL* and *psbA* genes. The presence of a 3′UTR derived from the different genes was required but had little effect on the accumulation of the transcript and foreign protein (Barnes et al., 2005). In later studies, the Goldschmidt-Clermont group demonstrated that the use of the psaA-exon1, its promoter and its 5′UTR increased foreign protein expression levels in the chloroplast. The authors also suggested that the variable expression observed among the different transformants that were derived from the same construct was related to the number and location of the recombination events (Michelet et al., 2011).

For a long time, foreign gene expression in the chloroplast suffered from low yields. With this in mind, limiting factors that could explain the low expression were extensively sought. It should be noted that gene expression in the chloroplast is tightly regulated at the translational level, and is less sensitive to transcriptional regulation. This was demonstrated in studies that inhibited the accumulation of specific chloroplast transcripts (by 90%) without affecting their translation rates. Gene dosage also proved to have little effect on the level of proteins encoded by these genes (Eberhard et al., 2002). The bottleneck in transgene expression from the chloroplast genome was addressed by the Mayfield group in 2007. They demonstrated that instead of targeting the expression cassette to the inverted repeats of the chloroplast genome, which yielded a low expression level of 0.5% relative to the total protein content in the cell, direct replacement of the *psbA* gene with a foreign coding region increased the production yield of the foreign protein to >5%. The authors suggested that the increased level of expression was due to a lack of competition encountered by regulatory factors (Mayfield and Schultz, 2004; Manuell et al., 2007).

Further improvements increased the level of transgene expression in the chloroplast of *C. reinhardtii* up to 20% of the total soluble protein content by focusing on optimization of codon usage and inhibition of ATP-dependent proteases. In addition, the toxicity of the transgene product may have a dramatic effect on its level of expression, and finally, large screens are usually required in order to isolate clones that efficiently express a transgene, possibly due to transformation-associated genotypic modifications that occur due to the random insertion of the foreign gene into the nuclear genome. Thus, the type of promoter and UTRs used are not the only factors that affect expression levels. The site of integration in the chloroplast, as well as accompanying random integration events in the nucleus, may also affect expression (Surzycki et al., 2009).

Chloroplast transformation systems that were developed for other algal species revealed differences with those developed for *Chlamydomonas*. For example, chloroplast transformation of *Euglena* revealed that the cassette containing the *aadA* gene flanked by the endogenous *psbA* promoter/5′UTR and 3′UTR generated drug resistant clones in which the DNA was maintained on a high molecular weight episomal element. Additional experiments using the *aadA* cassette introduced into the independently expressed *Euglena gracilis psbK* operon demonstrated proper splicing of the group III intron derived from the operon (Doetsch et al., 2001). Episomal elements carrying resistance genes are also common in non-photosynthetic kinetoplastids, such as *Leishmania* (Kapler et al., 1990), although the algal case describes a stable chloroplast episome.

### Inducible expression in the chloroplast

Inducible expression offers considerable advantages, especially when the product of the transgene may interfere with cell growth or be toxic. NAC2 is essential for stabilization of the *psbD* mRNA (Kuchka et al., 1989), binding a unique target site in the *psbD* 5′UTR, and regulating the expression of any foreign gene under control of the *psbD* 5′UTR. The group of Rochaix introduced the *NAC2* gene into *Chlamydomonas* cells under control of the cytochrome C_6_ promoter, which can be induced upon depletion of copper ions, by exposure to anaerobic conditions and in the presence of nickel ions (Merchant and Bogorad, 1987; Nickelsen et al., 1994; Quinn et al., 2002, 2003; Surzycki et al., 2007; Rochaix et al., 2014). Thus, manipulating the *NAC2* gene to be induced by any of these conditions resulted in a similar pattern of regulation of a foreign gene that was driven by the *psbD* 5′UTR (Rochaix et al., 2014).

## Nuclear transformation

Nuclear expression of transgenes in microalgae offers several advantages, including targeting of foreign proteins for expression in organelles, such as the chloroplast, and protein glycosylation and/or additional post-translational modifications, as well as secretion (León-Bañares et al., 2004). These advantages are especially required for the exploitation of microalgae for industrial production for recombinant proteins, especially in view of the known difficulties to obtain efficient expression of foreign genes in microalgae (Eichler-Stahlberg et al., 2009). A collection of protocols for nuclear transformation, combined with the availability of several constitutive or inducible promoters and the availability of multiple selectable markers, offers a multitude of approaches for expression of transgenes in the nucleus.

### Gene delivery methods

#### Generation of protoplasts

The algal cell wall represents a physical barrier preventing the entry of foreign DNA through the cell membrane. Thus, many protocols for transformation rely on the use of protoplasts, which are cell wall-deficient. However, in many cases, generating protoplasts is a major bottleneck due to the diverse composition of the cell wall in different algae species, which, in many cases, remain poorly characterized (Popper and Tuohy, 2010). The *Chlamydomonas* cell wall is built from glycoproteins and contains little cellulose or chitin. As such, polysaccharide-degrading enzymes are ineffective, with protoplasts instead being generated by incubation with autolysins, namely hydroxyl-proline-specific proteases that are active during gametogenesis and mating (Jaenicke et al., 1987; Imam and Snell, 1988). Unlike *Chlamydomonas*, the cell wall in *Chlorella* consists of sugar polymers that can be degraded by sugar digesting-enzymes (Takeda, 1991). Thus, protoplasts of different *Chlorella* species can be generated by incubation with different sugar-degrading enzyme mixes (Braun and Aach, 1975; Yamada and Sakaguchi, 1982; Afi et al., 1996). Protoplasts from red and brown algae have been prepared using numerous cell wall-digesting enzymes extracted from marine mollusks or echinoderms (Liu et al., 1984; Cheney et al., 1986; Reddy et al., 2010).

#### Biolistic delivery

The most frequently used method of gene delivery is biolistic transformation, also referred to as micro-projectile bombardment. This method utilizes DNA-coated gold or tungsten micro-particles that are delivered through a particle delivery system at high velocity into algal cells, surpassing the physiological barrier of the cell wall. Successful transformation by this approach have been reported for *C. reinhardtii* (Kindle et al., 1989), *Dunaliella salina* (Tan et al., 2005), *Haematoccucs pluvialis* (Steinbrenner and Sandmann, 2006), and diatoms (Dunahay et al., 1995; Apt et al., 1996; Falciatore et al., 1999; Zaslavskaia et al., 2000).

#### Glass beads method

A simple method that is often used for gene delivery is based on agitating protoplasts or cell wall-deficient mutants in the presence of glass beads, polyethylene glycol (PEG) and foreign DNA (Kindle et al., 1989). Successful transformations by this method were reported for *C. reinhardtii* (Kindle et al., 1989) and *Chlorella ellipsoidea* (Jarvis and Brown, 1991). In the case of *C. reinhardtii*, linearized plasmids usually yield higher transformation frequencies than supercoiled DNA when using glass beads or silicon carbide whiskers to mediate DNA entry (Kindle, 1990; Dunahay, 1993).

#### Electroporation

Applying an electric pulse is a commonly used method to introduce DNA into cells. This technique was used to transform microalgal protoplasts, cell wall-deficient mutants, and other thin walled algal cells. It was used to transform *C. reinhardtii* (Brown et al., 1991; Shimogawara et al., 1998; Yamano et al., 2013), *D. salina* (Walker et al., 2005b), *Chlorella vulgaris* (Chow and Tung, 1999), *Scenedesmus obliquus* (Guo et al., 2013), and *Nannochloropsis* sp. (Kilian et al., 2011). The use of cell wall-deficient strains improves transformation efficiency, with successful transformation using this methodology having been reported for *Lobosphaera* (Zorin et al., 2014). However, successful transformation without using cell wall mutants was also shown in *P. tricornutum*, either by electroporation (Niu et al., 2012; Zhang and Hu, 2014) or multi-pulse electroporation (Miyahara et al., 2013). In all these cases, the cells were grown without silica, which probably influenced cell wall structure. Overall, reducing cell wall thickness improves the transformation of microalga by electroporation.

#### *Agrobacterium*-based transformation of microalgae

The highly popular system for nuclear transformation of land plants using *Agrobacterium tumefaciens* was also adapted for transformation of microalga, using the pCAMBIA transformation vector. Several microalgal species were transformed by *Agrobacterium*, including *Chlamydomonas* (Kumar et al., 2004; Pratheesh et al., 2014), as well as other algae of biotechnological value, such as *H. pluvialis* (Kathiresan et al., 2015), *Schizochytrium* (Cheng et al., 2012), *Isochrysis galbana*, and *Isochrysis* sp. (Prasad et al., 2014).

### Nuclear promoters and control elements

#### Gene promoters

Efficient expression of foreign genes is achieved under the control of strong promoters. These are often derived from viruses, especially in land plants (Sanger et al., 1990), or from highly abundant endogenous genes, such as the small subunit of Rubisco (Goldschmidt-Clermont and Rahire, 1986). The use of a Cauliflower Mosaic Virus (CaMV35S) heterologous promoter that functions well in land plants gave inconsistent results when used to transform different algae species. Whereas, this promoter could drive the expression of reporter genes or chimeric genes in *D. salina* (Tan et al., 2005), *Chlorella kessleri* (El-Sheekh, 1999), and the dinoflagellates *Amphidinium* sp. and *Symbiodinium microadriaticum* (ten Lohuis and Miller, 1998; see Table 2) it gave contradictory results in *C. ellipsoidea* (Jarvis and Brown, 1991; Kim et al., 2002) and *C. reinhardtii* (Day et al., 1990; Blankenship and Kindle, 1992; Kumar et al., 2004; Diaz-Santos et al., 2013).

The use of strong endogenous promoters is recommended for nuclear transformation in algae, since heterologous promoters did not have an advantage, although they were active (Diaz-Santos et al., 2013). Among the endogenous regulatory elements, the *RBCS2* promoter that drives expression of the small subunit of Rubisco, and PSAD (an abundant chloroplast protein of the Photosystem I complex) from *C. reinhardtii* drove efficient expression of transgenes (Stevens et al., 1996; Lumbreras et al., 1998; Fuhrmann et al., 1999; Fischer and Rochaix, 2001). Improved expression of transgenes was demonstrated in *C. reinhardtii* when the *RBCS2* promoter was fused with the *HSP70A* (heat shock protein 70A) promoter, which acts as a transcriptional enhancer when placed upstream of the *RBCS2* promoter (Schroda et al., 2000). Driving expression by the *HSP70A/RBCS2* regulatory elements has become highly recommended for nuclear transformation of *Chlamydomonas*.

Other endogenous promoters have been used to drive the expression in other algal species, such as the promoter of the fucoxanthin-chlorophyll binding protein (*FCP*; Falciatore et al., 1999; Zaslavskaia et al., 2000; Poulsen et al., 2006). In the unicellular green alga *Lobosphaera (Parietochloris) incisa*, the endogenous *RBCS* promoter was used to drive expression of the *ble* gene, thus developing a platform for future successful engineering of this alga, which has great biotechnological potential due to its long-chain polyunsaturated fatty acid (PUFA) metabolism (Zorin et al., 2014).

Elements in the promoter region may occasionally have a negative effect on the expression of a transgene. Expression of the *ble* gene was silenced in 80% of transformants when it was driven from the *RBCS* promoter alone. However, when it was introduced under the control of the *HSP70A/RBCS* tandem promoter, silencing occurred in only 36% of transformed cells (Schroda et al., 2002). Modifying the *HSP70A/RBCS2* promoter so that it contained four copies of the first *RBCS2* gene intron between the *HSP70A* and *RBCS2* promoters significantly increased the expression of the downstream gene (Rasala et al., 2012).

A recent study described the use of the constitutive endogenous promoter that drives expression of the Glyceraldehyde-3-Phosphate Dehydrogenase (GAPDH), for expression of the bacterial gene encoding bialaphos resistance (*bar*) and of the *N*-terminal fragment of human canstatin in *D. salina* (Jia et al., 2012).

A search for efficient endogenous promoters was performed by a “promoter trapping approach” in *C. reinhardtii*, by which a promoter-less selectable marker gene was randomly integrated into the nuclear genome (von der Heyde et al., 2015). The appearance of drug-resistant colonies indicated that the selectable marker gene integrated near a strong promoter. This approach, combined with the growing amount of species whose genomes have been sequenced, should enable the isolation of strong endogenous promoters in other algae as well.

#### Inclusion of splicing signals

Another point of concern is the inclusion of signals for RNA processing in the algal nuclei, since only processed mRNAs can exit the nucleus through the nucleopores. Thus, expression cassettes that contain an endogenous intron were prepared for improvement of transgene expression (Lumbreras et al., 1998; Fischer and Rochaix, 2001; Kovar et al., 2002).

#### Optimization of codon usage

The absence of tRNAs that are compatible with the codon usage of a given gene can dramatically affect expression of a transgene (Heitzer et al., 2007). Differences in codon usage were recorded between various microalgal species, as well as for the chloroplast and nuclear genomes of the same species (León-Bañares et al., 2004) and between organelles (Jarvis et al., 1992). Therefore, codon optimization of exogenous genes can significantly improve protein expression (Zaslavskaia et al., 2000; Franklin et al., 2002).

### Inducible expression in the nucleus

The biotechnological exploitation of constitutive promoters for the effective expression of toxic compounds for industrial or pharmaceutical uses or for establishing silencing systems may occasionally be problematic. Hence, introducing inducible expression systems offers considerable advantages. Overall, the use of inducible systems is also attractive for large-scale production of recombinant proteins in microalgae, as these enable the cells to first reach an optimal concentration before expression of the transgene is initiated.

Nitrogen metabolism is based on the exploitation of ammonium ions. However, some algae can adsorb nitrate and convert it into ammonium with the help of nitrate reductase (Fernandez et al., 1989; Berges, 1997). The promoter of the nitrate reductase gene that is switched on and off in response to the presence of nitrate or ammonium ions (Poulsen and Kroger, 2005) was able to promote the inducible expression of a reporter gene in *Cylindrotheca fusiformis* (Poulsen and Kroger, 2005), *C. reinhardtii* (Schmollinger et al., 2010), *C. ellipsoidea* (Wang et al., 2004), *D. salina* (Li et al., 2007, 2008), *Volvox carteri* (von der Heyde et al., 2015), and *Phaeodactylum triornutum* (Niu et al., 2012).

The use of light for inducing expression in microalgae has long been pursued, initially by the use of the *CABII-1* promoter that was shown to respond to changing light conditions and stimulate expression of the *NIT1* gene under light in *Chlamydomonas* (Blankenship and Kindle, 1992). More recently, the light-regulated elements of the light-inducible protein *(LIP)* from *Dunaliella* was mapped to the flanking 400 bp upstream sequences of the *LIP* gene, which contains triplicates of light-responsible motifs. These were shown to induce expression of a heterologous reporter gene in *Chlamydomonas* (Park et al., 2013; Baek et al., 2016).

The metal-responsive *CYC6* promoter that is repressed by copper was used for inducible gene expression in *C. reinhardtii* (Quinn and Merchant, 1995). The copper response element (*CuRE*) is responsive to both nickel and cobalt ions, and could be inhibited by EDTA (Quinn et al., 2003). Expression from this inducible system was improved by the addition of the first intron of the *RBCS2* gene to the *CYC6* promoter (Ferrante et al., 2011). In several marine and freshwater algae species, a control element located upstream of the B_12_-independent methionine synthase (*METE*) gene could repress the expression of a reporter gene following addition of B_12_ (Helliwell et al., 2014). The strong repressible nature and high sensitivity of the B_12_-responsive element can, therefore, be used as another promising gene expression tool for biotechnological applications.

Yet another element that was used to drive transgene expression is based on riboswitch biology (Mandal and Breaker, 2004). A riboswitch-regulated element based on the thiamine pyrophosphate (TPP) biosynthesis was identified in the *THIC* genes of a variety of organisms (Breaker, 2012), including *Arabidopsis*, where it affected *THIC* mRNA stability by alternative splicing of the primary mRNA (Bocobza et al., 2007; Wachter et al., 2007; Bocobza and Aharoni, 2014). This TPP riboswitch was later used for generating an inducible expression system in *Arabidopsis* (Bocobza et al., 2013) and in *C. reinhardtii* (Ramundo et al., 2013). Using the TPP system, Ramundo and colleagues were able to conditionally repress expression of the *rpoA* or *rps12* genes in the chloroplast, affecting their organelle transcription or translation, respectively. Although riboswitch elements are common in prokaryotes, and have been used to generate inducible expression systems in bacteria (Neupert and Bock, 2009), the only riboswitch to be identified in higher eukaryotes was the TPP riboswitch, described above. The use of riboswitch-based regulation was recently used to develop a system for increasing expression of chloroplast genes in *Arabidopsis*, via an RNA amplification-based system that strongly improves the efficiency of riboswitches (Emadpour et al., 2015). A summary of the constitutive and inducible expression systems is given in Table 2.

### Nuclear selectable marker genes

Stable transformation is based on the use of a proper selection marker. These include genes that confer resistance to antibiotics or herbicides, and various metabolic enzymes that control growth under specific nutritional conditions. A collection of nuclear selective genes are listed in Table 3.

**Table 3:** **List of selection markers and selection modes for nuclear transformation**.

**Type of selection marker**	**Selection gene**	**Gene product**	**Selection mode**	**Species/Genetic background**	**References**
Antibiotic resistance	*Ble*	Phleomycin-binding protein	Resistance to Zeocine/Phleomycin	*Chlamydomonas reinhardtii*	Stevens et al., 1996; Guo et al., 2013
				*Dunaliella salina*	Sun et al., 2005
				*Volvox carteri*	Hallmann and Rappel, 1999
				*Chlorella ellipsoidea*	Kim et al., 2002
				*Phaeodactylum tricornutum*	Apt et al., 1996; Falciatore et al., 1999; Zaslavskaia et al., 2001; De Riso et al., 2009; Kira et al., 2015
				*Cylindrotheca fusiformis*	Fischer et al., 1999; Poulsen and Kroger, 2005
				*Nannochloropsis* sp.	Kilian et al., 2011
				*Nannochloropsis granulate/gaditana/oculata/ oceanica/salina*	Li et al., 2014
	*aphVIII*	Aminoglycoside 3′-phosphotransferase	Resistance to Paromomycin	*Chlamydomonas reinhardtii*	Sizova et al., 2001
				*Gonium pectoral*	Lerche and Hallmann, 2009
				*Eudorina elegans*	Lerche and Hallmann, 2013
	*aadA*	Aminoglycoside 3′-adenylyltransferase	Resistance to Spectinomycin/Streptomycin	*Chlamydomonas reinhardtii*	Cerutti et al., 1997b
	*aph7*	Aminoglycoside phosphotransferase	Resistance to Hygromycin	*Chlamydomonas reinhardtii*	Berthold et al., 2002
				*Haematococcus pluvialis*	Kathiresan et al., 2015
				*Volvox carteri*	Jakobiak et al., 2004
				*Chlorella vulgaris*	Chow and Tung, 1999
				*Laminaria japonica*	Qin et al., 1999
	*nptII*	Neomycin phosphotransferase	Resistance to Neomycin	*Chlamydomonas reinhardtii*	Hall et al., 1993
				*Chlorella sorokiniana, Chlorella vulgaris*	Hawkins and Nakamura, 1999
				*Amphidinium* sp., and *Symbiodinium microadriaticum*	ten Lohuis and Miller, 1998
				*Cyclotella cryptica, Navicula saprophila*	Dunahay et al., 1995
				*Phaeodactylum tricornutum*	Zaslavskaia et al., 2000
	*cat*	Chloramphenicol acetyltransferase	Resistance to Chloramphenicol	*Dunaliella salina*	Geng et al., 2004; Sun et al., 2008
				*Chlorella vulgaris*	Niu et al., 2011
	*CRY1-1*	Cytosolic ribosomal protein S14	Resistance to Emetine	*Chlamydomonas reinhardtii*	Nelson et al., 1994; Neupert et al., 2009
Herbicide resistance	*GAT*	Glyphosate aminotransferase	Resistance to Glyphosate	*Chlamydomonas reinhardtii*	Bruggeman et al., 2014
	*ALS*	Acetolacetate synthase	Resistance to Sulfometuron methyl	*Chlamydomonas reinhardtii*	Kovar et al., 2002
				*Porphyridium* sp.	Lapidot et al., 2002
				*Parietochloris incisa*	Grundman et al., 2012
	*PDS1*	Phytoene desaturase	Resistance to Norflurazon	*Haematoccucs pluvialis*	Steinbrenner and Sandmann, 2006; Sharon-Gojman et al., 2015
				*Chlorella zofingiensis*	Huang et al., 2008; Liu et al., 2014
Metabolic markers	*NIT1*	Nitrate reductase	Growth in the presence of nitrate salt	*Chlamydomonas reinhardtii* (*nit1*-)	Kindle et al., 1989
				*Volvox carteri* (*nit1*-)	Schiedlmeier et al., 1994; Hallmann and Sumper, 1996
				*Dunaliella viridis* (*nit1*-)	Sun et al., 2006
				*Chlorella sorokiniana* (*nit1*-)	Dawson et al., 1997
				*Chlorella ellipsoidea* (*nit1*-)	Bai et al., 2013
	*ARG7*	Argininosuccinate lyase	Growth in Arginine free media	*Chlamydomonas reinhardtii* (*arg7*-)	Debuchy et al., 1989; Haring and Beck, 1997; Molnar et al., 2009
	*NIC7*	Quinolinate synthetase	Growth in Nicotinamide free media	*Chlamydomonas reinhardtii* (*nic7*-)	Ferris, 1995; Adam et al., 2006; Lin et al., 2010
	*OEE1*	Oxygen-evolving enhancer protein1	Photoautotrophic growth	*Chlamydomonas reinhardtii* (*oee1-*)	Mayfield and Kindle, 1990

#### Selection of nuclear transformants based on auxotrophic growth

Auxotroph genes can be used as selection markers for rescuing the phenotype of specific mutants that restrict growth under minimal conditions. The *NIT1* gene, encoding nitrate reductase, promotes growth in the presence of nitrates as a nitrogen source (Fernandez et al., 1989), and was introduced for the nuclear transformation of the *NIT1* mutant of *C. reinhardtii* (Kindle et al., 1989). This useful selection system was also applied for many other algal species (see Table 3). Another metabolic marker is the *ARG7* gene, encoding argininosuccinate lyase. It was used to rescue arginine-requiring *ARG7* mutants to prototrophy (Debuchy et al., 1989; Haring and Beck, 1997), and is commonly used for nuclear transformation of *C. reinhardtii*. The ARG7 mutant strain has been widely used for transformation of *Chlamydomonas*, mostly for basic research. A similar approach for use with other algae would be welcome, although a mutant that does not express argininosuccinate lyase must first be isolated. Another selection marker is based on *NIC7*, encoding quinolinate synthetase that is required for NAD biosynthesis. A plasmid carrying this gene could rescue a mutation in the *C. reinhardtii NIC1* gene (Ferris, 1995; Lin et al., 2010), allowing growth in the absence of nicotinamide.

#### Selection of nuclear transformants based on resistance to antibiotics

As with animal cell systems, numerous antibiotics genes have been successfully used as selection markers of microalgae. The *ble* gene that was originally isolated from *Streptoalloteichus hindustanus*, confers resistance to zeomycin and phleomycin (Sugiyama et al., 1994) and was used for generating transgenic clones of different algal species (see Table 3). The synthetic aminoglycoside adenyltransferase *aadA* gene confers resistance to spectinomycin and streptomycin (Svab and Maliga, 1993). It was originally used for chloroplast transformations but was further adopted for the nuclear system in *Chlamydomonas* (Cerutti et al., 1997b), and *H. pluvialis* (Gutiérrez et al., 2012). The aminoglycoside phosphotransferase genes *aphVIII* (*aphH)* from *Streptomycesrimosu*s and *aph7* from *Streptomyces hygroscopicus* confer resistance to paromomycin (Sizova et al., 2001) and hygromycin B, respectively. These were used to select drug-resistant algae in several species (see Table 3). The bacterial *nptII* gene encoding neomycin phosphotransferase was proven to be relatively inefficient in *C. reinhardtii* (Bingham et al., 1989; Hall et al., 1993). Codon optimization of the *nptII* gene, however, led to its improved function as a nuclear selection marker (Barahimipour et al., 2016). This selection marker was more successful when used for transforming other algal strains (see Table 3), as well as seed plants (Elghabi et al., 2011). The use of the *cat* gene, encoding CAT that confers resistance to chloramphenicol, has also been widely used (Table 3). Finally, the mutated version of the *C. reinhardtii* gene encoding ribosomal protein S14 (*CRY1*) confers resistance to emetine and cryptopleurine, and was adopted as a dominant selection marker (Nelson et al., 1994). Indeed, most antibiotic resistance genes have been successfully and routinely used for algal transformations (see Table 3). Nonetheless, their use as selection markers raises both health and ecological concerns for large-scale production in plants and microalgae.

#### Selection of nuclear transgenic algae based on herbicide resistance

The demand for production of plants and microalgae that are free of antibiotic resistance markers encouraged the use of herbicide-resistance markers, such as acetohydroxyacid synthase (*ALS/AHAS*) that confers resistance to sulfometuron methyl (SMM), phytoene desaturase (*PDS*) that generates resistance to the bleaching herbicide norflurazon, and glyphosate acetyltransferase (*GAT*) that contributes to resistance against glyphosate (Malik et al., 1989).

ALS/AHAS catalyzes the first step in the biosynthesis of the branched-chain amino acids, valine, leucine, and isoleucine (Kishore and Shah, 1988; Chipman et al., 1998). It is the target enzyme of the SMM herbicide that effectively inhibits growth of bacteria, yeast, plants, and algae. A mutant form of the gene encoding ALS/AHAS was used as a dominant selectable marker in the green algae *Lobosphaera (Parietochloris) incisa* (Grundman et al., 2012), based on experience gained from transformation of the chloroplast of the unicellular red alga *Porphyridium* sp. (Lapidot et al., 2002), and the green algae *C. reinhardtii* (Kovar et al., 2002).

PDS functions in the carotenoid biosynthesis pathway. Inhibition of this enzyme causes degradation of chlorophyll and the chloroplast membrane, as well as photo-bleaching of green tissues (Boger and Sandmann, 1998). Point mutations in *PDS* confer enhanced resistance to the bleaching herbicide norfurazon in the green microalgae *H. pluvialis* (Steinbrenner and Sandmann, 2006; Sharon-Gojman et al., 2015) and *Chlorella zofingiensis* (Huang et al., 2008; Liu et al., 2014). The use of GAT that confers increased tolerance to glyphosate is less recommended due to the reduced growth of *GAT*-expressing transformants in response to high concentrations of glyphosate, which were required to inhibit wild type cells (Bruggeman et al., 2014).

#### Targeting of foreign proteins to the chloroplast

Targeting foreign proteins to sub-cellular compartments can affect their expression yield through protein folding, assembly and post-translational modifications. For example, the chloroplast provides an oxidizing environment that promotes the formation of disulfide bridges, and an abundance of chaperones that are required for the folding of soluble proteins after their import. The ability to properly fold and generate native disulfide bridges is fundamental for generating functional proteins and protein complexes, thus making the chloroplast an attractive organelle for the expression of recombinant proteins. This was demonstrated in *C. reinhardtii* by the production of the disulfide-containing Pfs25 protein from *Plasmodium falciparum*, which blocks transmission of the malaria parasite (Gregory et al., 2012). The importance of disulfide bond formation was also demonstrated by monitoring the expression and assembly of several fully active antibodies against pathogenic agents (Mayfield et al., 2003; Mayfield and Franklin, 2005).

Another advantage of organelle-specific expression is that targeting of nuclear-expressed proteins to chloroplasts can reduce the danger of proteolysis (Barnes et al., 2005; Doran, 2006; Mayfield et al., 2007). However, it still remains unclear why the ability to accumulate high levels of exogenous proteins in the chloroplast is heterogeneous. The plastid contains three large families of proteases, each of bacterial origin. These include the ATP-dependent Zn-metallo protease FtsH family (Adam et al., 2006; Liu et al., 2010), the ATP-independent Deg/HtrA family of serine endopeptidases (Huesgen et al., 2009; Sun et al., 2010) and the ATP-dependent serine-type Clp family (Adam et al., 2006). The chloroplast proteases can cleave exogenous proteins, as demonstrated in *C. reinhardtii* by the use of cyanide m-chlorophenylhydrazone (CCCP), which uncouples chloroplast and mitochondrial energy production. The presence of CCCP reduced degradation of the model protein VP28 from the White Spot Syndrome Virus three-fold expressed in the chloroplast, demonstrating that ATP-dependent proteases are involved in degrading this protein (Surzycki et al., 2009). Most chloroplast proteases are encoded in the nucleus, except for ClpP1. Thus, attempts to limit proteolysis of foreign genes expressed in the chloroplast can be controlled by knockdown technologies, such as RNAi, an approach that still requires further improvement in microalgae. Attempts to reduce expression of chloroplast-encoded ClpP by riboswitch control resulted in malfunctioning cells (Ramundo et al., 2014). However, deletion of the gene encoding ClpP protease from the chloroplast was achieved in *A. thaliana* (Zheng et al., 2006; Stanne et al., 2009), indicating that this approach could be feasible for microalgae as well.

Protein toxicity should also be considered when expression of foreign proteins in photosynthetic organisms is attempted. For example, the toxic effect of avidin, when expressed in the cytosol of transgenic tobacco plants, can be surpassed when this molecule is targeted to the vacuole (Murray et al., 2002). This also holds true for the cholera toxin-B subunit that is toxic to tobacco cells when expressed in the cytosol but not in the chloroplast (Daniell et al., 2001). Similar aspects should be considered when expressing foreign genes in microalgae.

Attempts to identify a consensus sequence shared by chloroplast and mitochondrial targeting peptides (cTPs and mTPs, respectively) were only partially successful (Habib et al., 2007; Huang et al., 2009). For this reason, data-driven machine learning techniques were developed (Schneider and Fechner, 2004), leading to the development of several programs, such as TargetP and Predotar (Emanuelsson et al., 2000; Emanuelsson, 2002; Small et al., 2004), that try to predict targeting peptides (TPs) for land plants. ChloroP is another program that predicts cleavage sites of TPs (Emanuelsson et al., 1999). These programs are based on the detection of an N-terminal targeting sequence that is shared between different targeted polypeptides. However, since green algae diverged from land plants over 725–1200 million years ago (Becker and Marin, 2009), their organelle import machineries, as well as their TPs, differ substantially from those of land plants. As such, most prediction programs are less reliable when used to predict the localization of algal proteins (Patron and Waller, 2007). With this in mind, a new algorithm, PredAlgo, that identifies cTPs/mTPs in green algae was developed. It is based on the accumulation of a large dataset from large- and small-scale proteomic studies of *Chlamydomonas* organelles (https://giavap-genomes.ibpc.fr/cgi-bin/predalgodb.perl?page=main; Tardif et al., 2012).

A recent study, based on the genomes of *P. tricornutum* and *Thalassiosira pseudonana*, provides a detailed analysis of TP motifs that target nuclear-encoded proteins to chloroplasts in diatoms and algae with secondary plastids of the red lineage (i.e., dinoflagellates, cryptophytes, and stramenopiles; Gruber et al., 2015). Organisms that belong to these phyla are of great interest to algae-based industry.

#### Targeting transgene products for expression in specific organelles or secretion

Targeting a transgene protein product for secretion is a common strategy for avoiding its degradation. A dedicated vector was designed in which luciferase expression in *C. reinhardtii* was greatly improved (up to 84%) by fusing the transgene with the previously identified secretion element of carbonic anhydrase (Lauersen et al., 2013a). This approach was further implemented for the production of secreted ice-binding protein, a protein of considerable industrial value (Lauersen et al., 2013b, 2015).

The targeting of nuclear transformation proteins to different cellular compartments is achieved by the addition of a TP-encoding sequence at the 5′-end of the transgene. TPs are recognized by the import machineries, which direct import into the proper organelle. Vectors that efficiently and specifically target transgene products to different compartments were generated (Rasala et al., 2014). Such vectors introduce TPs to the nucleus, mitochondria, chloroplast and ER using the nuclear localization signal (NLS) from simian virus 40, the N-terminal mTP from the nuclear gene encoding the alpha subunit of the mitochondrial ATP synthase, the N-terminal cTP from the photosystem I reaction center subunit II (encoded by p*saD*), and the ER-transit sequence from either BiP1 or ARS1, respectively. The TP that targets proteins to peroxisomes was also identified and was further shown to target a GFP transgene to the peroxisome of *C. reinhardtii* (Hayashi and Shinozaki, 2012).

In some cases, protein tagging is required for downstream applications, such as pull-down assays, immunoprecipitation, or protein purification. The introduction of such tags could interfere with the targeting signals. To prevent the obscuring of subcellular targeting signals, it is important to identify the TP of the native protein. In *Chlamydomonas*, endogenous proteins are usually tagged at their C-termini, since TPs are most frequently found at the N-terminus (Franzen et al., 1990; Patron and Waller, 2007).

### Increasing the efficiency of transgene nuclear expression

While chloroplast transformation toolkits have been established for several microalgae, similar tools for nuclear-based protein expression remain under-developed, as for land plants. The reasons that lead to the low expression of transgenes from the nuclear genome could be varied, including the effects of position on integration events, epigenetic-derived transgene silencing, and difficulties related to variable codon usage systems (Jinkerson and Jonikas, 2015). Although the precise mechanism of insertion of DNA into the genome is unknown, it involves ligation of the transforming DNA at a site of double-stranded genomic DNA break, an event that occurs randomly throughout the genome with little sequence specificity, via the non-homologous end joining repair pathway (Kindle et al., 1989; Mayfield and Kindle, 1990; Zhang et al., 2014). In some cases, the transformed DNA integrates as a whole cassette, although truncated versions, fragments, or multiple cassettes that result from enzymatic cleavage can also be inserted (Zhang et al., 2014). Random integration events can sometimes result in “position effects,” in which the level of transgene expression is influenced by the surrounding genomic regions (Leon and Fernandez, 2007). It is generally accepted that screening of a large number of transformants in search of a high expressing clone is necessary (Hallmann, 2007).

#### Generation of strains for improved transgene expression

Despite the great advances made in developing systems for algal transformation, with *C. reinhardtii* serving as a fully sequenced model organism (Blaby et al., 2014), transgene expression using different methodologies and approaches remained limited until specific mutant strains capable of increased expression were isolated (Neupert et al., 2009). The basic assumption was that transgene expression could be affected by epigenetic processes, although the exact reasons remained unclear. To overcome this, a genetic screen was established in which wild type *C. reinhardtii* cells were subjected to random mutagenesis, and further screened for colonies that showed increased expression of the transgene. The screen used the ARG^−^ selection system to select for transgenic algae, although another level of selection was introduced to screen for clones with increased expression. This was achieved by introducing *CRY1-1*, a ribosomal gene that confers resistance to emetine in a dose-responsive manner. As such, clones with increased *CRY1-1* expression could grow in the presence of high emetine concentrations. Two clones, UVM4 and UVM11, were selected for increased transgene expression and are currently used for in *Chlamydomonas* (Lauersen et al., 2013b). It is tempting to examine whether a parallel system could be developed for other algae as well.

#### Improvement of transgene expression by fusion with a selection marker

An expression cassette in which the codon-optimized *GFP* gene was fused to the *ble* selection marker increased GFP expression in *C. reinhardtii* (Fuhrmann et al., 1999). A further improvement was achieved by including the self-cleavable 2A peptide derived from the foot and mouth disease virus (Ryan et al., 1991) between the transgene and the *ble* selection marker. Its presence resulted in processing of the fused polypeptides to yield two independent proteins. This system led to an ~100-fold increase in the expression of several transgenes (Rasala et al., 2012, 2013).

The combination of using novel algal strains that promote increased expression of foreign genes, along with the sophisticated fusion between selection and target genes, opens a new era in transgene expression by the green algae *Chlamydomonas*, although similar developments with other algae are called for as well. The use of *Chlamydomonas* overexpressing strains (i.e., UVM11), combined with the codon optimization of the target gene, was shown to overcome the expression barrier of transgenes in *Chlamydomonas*. This approach led to the efficient expression of an HIV vaccine candidate, P24 (Barahimipour et al., 2016).

#### Expression of transgenes from episomes

A recent study reported the development of a nuclear episomal vector designed to introduce foreign DNA from *E. coli* into two diatom species, *P. tricornutum* and *T. pseudonana*, via conjugation. The vector contained a yeast-derived sequence that promoted its replication in these diatoms, even after antibiotic selection was eliminated. This episome was maintained as a closed circle at a copy number equivalent to the number of native chromosomes (Karas et al., 2015). This system offers great advantages in that it offers an easy method for introducing large DNA fragments into the host microalga, possibly promoting entry of several genes comprising a metabolic pathway. In addition, it is expected, although has yet to be shown, that expression of foreign genes from an episome would be less interrupted by epigenetic mechanisms or positional effects, since the DNA is not integrated into the chromosomal genome.

### Gene targeting in algal nuclei

Nuclear transformation of microalgae, in most studied cases, exhibited low frequency of homologous recombination (Sodeinde and Kindle, 1993; Nelson and Lefebvre, 1995), as was also observed with most photosynthetic organisms, except for mosses. Although occasional successful homologous recombination in the nuclei of *Chlamydomonas* had been reported in the past (Sodeinde and Kindle, 1993; Nelson and Lefebvre, 1995; Dawson et al., 1997; Hallmann et al., 1997; Minoda et al., 2004; Zorin et al., 2005), its frequency was too low for adaptation as a recommended technology. Efficient homologous recombination suitable for biotechnology applications was recorded in only two eukaryotic algae, namely *Cyanidioschyzon merolae* (Minoda et al., 2004) and *Nannochloropsis* sp. (Kilian et al., 2011).

Attempts to overcome difficulties that arise from positional effects and/or random integration of transgenes included the development of a Zn-finger nuclease system (Townsend et al., 2009) that can recognize and cleave a relatively long specific sequence, generating site-specific double-strand DNA breaks, thus modifying a gene of interest, as shown for the *COP3* gene encoding a light-activated ion channel (Sizova et al., 2013). Another methodology that is currently being developed for microalgae is based on the CRISPR/Cas9 system, which uses a guide RNA that directs the Cas9 nuclease to restrict a specific sequence of DNA (Jinek et al., 2012). Components of the CRISPR/Cas9 system have recently been shown to function in a transient manner in *C. reinhardtii*, although the high toxicity of Cas9 prevented the successful recovery of stable colonies (Jiang et al., 2014). As such, further improvement of the CRISPR/Cas9 system is required.

Nuclear transgenic expression in microalgae is occasionally inefficient, possibly due to gene silencing (Cerutti et al., 1997a), or other reasons that are not yet fully understood. Still, even if all the elements required for optimal transcription and translation of transgene are provided, expression of exogenous genes can be very low or even non-existent, possibly due to gene silencing (Cerutti et al., 2011). Furthermore, expression of exogenous genes might be eliminated if transgenic algae clones are not maintained under constant selection conditions.

### Gene silencing in algae through the RNAi machinery

The recent sequencing of several algal genomes has provided insight into the great complexity of these species, although algal physiology and metabolomics are still not fully resolved. Nonetheless, key components of the RNA-mediated silencing machinery, such as Dicer and Argonaute that can process double-stranded RNA (dsRNA) into small interfering RNAs (siRNAs; Sontheimer and Carthew, 2005; Ghildiyal and Zamore, 2009; Voinnet, 2009; Fabian et al., 2010), have been found in many algae species, including *C. reinhardtii* (Schroda, 2006; Merchant et al., 2007; Kim and Cerutti, 2009), *V. carteri* (Ebnet et al., 1999; Cheng et al., 2006; Prochnik et al., 2010), *D. salina* (Sun et al., 2008; Jia et al., 2009), *P. tricornutum* (Bowler et al., 2008; De Riso et al., 2009), and *E. gracilis* (Iseki et al., 2002; Ishikawa et al., 2008). RNA-mediated silencing pathways have been studied in the unicellular green alga *C. reinhardtii*, where they are used as a reverse genetics tool for targeted knockdown of a variety of genes (Sineshchekov et al., 2002; Rohr et al., 2004; Soupene et al., 2004; Schroda, 2006). RNAi methodology was also used to examine the function of Aureochrome, a photoreceptor required for photomorphogenesis in stramenopiles. Silencing of the *AUREO2* gene by introduction of dsRNA derived from the target gene induced the formation of sex organ primordia instead of branches, implicating it in the initiation of the development of a branch but not of a sex organ (Takahashi et al., 2007).

RNA-mediated silencing pathways have been studied in the unicellular green alga *C. reinhardtii* and used as a reverse genetics tool for targeted knockdown of a variety of genes (Sineshchekov et al., 2002; Rohr et al., 2004; Soupene et al., 2004; Schroda, 2006). The use of artificial miRNAs (amiRNAs), which mimic the structure of endogenous miRNA precursors was designed to enhance this approach (Molnar et al., 2009; Zhao et al., 2009). AmiRNAs can be designed by the microRNA designer platform (WMD3, http://wmd3.weigelworld.org/cgi-bin/webapp.cgi, active on March 14, 2016), which identifies suitable amiRNA candidates, based on optimal and specific hybridization properties, to target mRNA (Ossowski et al., 2008). Stable RNAi gene silencing was demonstrated in different microalgae as well, including *V. carteri* (Ebnet et al., 1999; Cheng et al., 2006), *D. salina* (Jia et al., 2009), *P. tricornutum* (De Riso et al., 2009), and *E. gracilis* (Iseki et al., 2002). Non-integrative dsRNA or siRNA was also used to trigger temporary RNAi in the green alga *D. salina* (Sun et al., 2008), *E. gracilis* (Ishikawa et al., 2008), and *Vaucheria frigida* (Takahashi et al., 2007).

## Biotechnological exploitation of microalgae

Microalgae offer substantial potential for various biotechnological purposes, including production of animal food, aquaculture and marine agriculture, and biosynthesis of medical products, such as oral vaccines for animals (fish) and humans, high-value nutritional additives (e.g., polyunsaturated fatty acids, carotenoids, etc.), food dyes, and compounds used in the cosmetic industry and various other chemicals, in addition to possible future uses as a sources of energy. Moreover, microalgae-based technologies can be developed into water source treatments. These highly attractive goals justify the great efforts that the scientific community is investing in the development of molecular tools for the generation of transgenic microalgae.

### Using synthetic biology for the production of commercial added value in algae

In view of the difficulties in obtaining a high level of transgene expression in the cytoplasm of algae, the production of numerous therapeutic proteins was targeted to the chloroplast, including antibodies and proteins for use as oral vaccines. For example, a single-chain antibody was produced against the herpes simplex virus glycoprotein D (Mayfield et al., 2003; Mayfield and Franklin, 2005), and an IgG1 monoclonal antibody was generated against the anthrax protective antigen 83 (Tran et al., 2009). There were also attempts to use the chloroplast for producing oral vaccines against malaria (Gregory et al., 2012, 2013; Jones et al., 2013; Patra et al., 2015), *Staphylococcus aureus* (Dreesen et al., 2010), the white spot syndrome virus protein 28 (Surzycki et al., 2009), the P57 antigen bacterial kidney disease, (Siripornadulsil et al., 2007), and the VP1 antigen of foot and mouth disease virus fused to the Cholera toxin B subunit (Sun et al., 2003). Microalgae were exploited for the production of other immune reactive proteins, such as the human glutamic acid decarboxylase, a known auto-antigen in type 1 diabetes (Wang et al., 2008).

Heterologous therapeutic proteins were expressed not only in the chloroplast, but also in the nuclei of different algae, such as *D. salina, Porphyridium* sp., *Nannocholorpsis oculata*, and *C. reinhardtii*, to produce edible vaccines against the malaria parasite *Plasmodium berghei* (Dauvillée et al., 2010), the surface antigen of Hepatitis B virus (Geng et al., 2003) and for the production of food additives such as the xylanase growth hormone (Rasala et al., 2012; Georgianna et al., 2013) and Sep15, a selenium supplement (Hou et al., 2013). Algal-derived oral vaccines were shown to have a long shelf-life and their administration is injection-free. However, as the use of oral vaccines elicits mainly local immunity, their general efficacy must be carefully monitored.

Microalga have been harnessed to express enzymes of interest (Rasala et al., 2010, 2012), as well as for the potential production of biofuels (Georgianna and Mayfield, 2012), although the economical validity of this approach is still not satisfactory. Efforts were also targeted to enhancing the metabolic engineering of algae for enrichment of lipids, although this goal has yet to be reached (Dunahay et al., 1996).

Genetic manipulations could also help in adapting microalgae to different growth conditions. Attempts to convert autotrophic algae into heterotrophs relied on introducing the *HUP1* gene form *C. kessleri*, which encodes for the hexose/H+ transporter that enables growth in the dark, into *V. carteri* (Hallmann and Sumper, 1996) and *P. tricornutum* (Zaslavskaia et al., 2001). Increased hydrogen production (by 150%) was obtained when the *C. reinhardtii* SMT6 mutant, which is a high H_2_-producing strain, was transformed with the *HUP1* gene. This study established the possibility of improving the production of H_2_ from H_2_0 and glucose (Doebbe et al., 2007). Increased hydrogen production was also obtained by a triple knockdown of the light harvesting complex proteins LHCMB1, 2 and 3 by RNAi, which resulted in a 180% increase in hydrogen production (Oey et al., 2013).

Cartenoid production from *D. salina* and *H. pluvialis* is also of great commercial interest, and is based on the natural production of β-carotene and astaxanthin. Manipulation of these species is possible, with increased expression of phytoene desaturase resulting in accelerated biosynthesis of astaxanthin. Production of an L504R mutant of phytoene desaturase resulted in increased production of astaxanthin (Steinbrenner and Sandmann, 2006). Furthermore, introducing *the C. zofingiensis* phytoene synthase, an enzyme that participates in the carotenoid biosynthetic pathway, into *C. reinhardtii* resulted in increased levels of violaxanthin and lutein (Cordero et al., 2011). These studies emphasize the great potential in manipulating metabolic pathways of high value products by engineering different algal species. Overall, microalgal systems hold great biotechnological potential that will surely be exploited in the future.

### Biosafety considerations

In the European Union, all genetically modified organisms (GMOs) or their products must receive approval before they can find their way to market. This practice also applies to genetically modified (GM) microorganisms. Thus, a guidance protocol for risk assessment of genetically modified microorganisms was prepared by the European Food Safety Authority (EFSA) in 2006 (http://www.efsa.europa.eu/sites/default/files/scientific_output/files/main_documents/374.pdf). The guiding protocols discriminate between the contained use of microalgae and their deliberate release into the environment, as further discussed in an OECD meeting on the Biosafety and Environmental Uses of Micro-Organisms (Wijffels, 2015). Contained use refers to closed conditions in which GM microorganisms are grown, stored, transported, destroyed and disposed, whereas deliberate release refers to GM microorganisms that are released in the environment without restricting their ability to spread. Contained growth can be performed in closed bioreactors (Chaumont, 1993), tubular reactors (Richmond et al., 1993), or polyethylene sleeves (Cohen et al., 1991). These technologies are designed to allow efficient growth and the harvesting of large quantities of microalgal biomass. Examples of deliberate release of GM microorganisms include their use for immunization of fish against various pathogens in open ponds, or when microalgae are employed for cleaning polluted water sources. Risk assessment is usually performed by testing the effects a GM microorganism has on humans, animals, plants and/or the environment, and comparing these effects with those of the conventional non-transformed variant. Risk assessment refers to the toxicity and allergenicity of the GM microorganism or its products, as well as the potential risk of horizontal gene transfer (HGT). To date, there are only a few reports on HGT in microalgae, and these refer mainly to viral-mediated HGT (Monier et al., 2009; Rosenwasser et al., 2014). However, to address this potential risk, even if it is very rare, the use of selection modes based on endogenous metabolic markers that complement a missing activity in a mutant recipient strain would be advantageous over the use of genes that confer resistance to herbicides or antibiotics. Removal of the selection markers is also recommended.

## Conclusions and future perspectives

Despite occupying the base of the plant evolutionary tree, algae are distributed worldwide and grow in different climates, under normal and extreme conditions. As such, algae are known to provide a rich repertoire of chemicals and bio-molecules, including those of interest to biotechnological industries. Algae can also be harnessed as an important food source in the frame of aquatic agriculture. Finally, they provide a powerful resource for the production of bio-medical molecules, such as vaccines, antibodies and drugs (Cadoret et al., 2012). Another attractive achievement will enable the introduction of foreign genes that comprise a metabolic pathway that generates a valuable compound. A recent effort to express the complete carotenoid pathway toward enhanced astaxanthin formation was reported for *Xanthophyllomyces dendrorhous* (Gassel et al., 2014). However, these exciting possibilities are fully dependent on efficient expression of transgenes in microalgae. Indeed, this bottleneck must be addressed, since for occasional cases transgene expression had proven to be rather inefficient, for reasons that were not fully understood. Foreign genes can be targeted for expression in the chloroplast either by integration into the chloroplast genome or by their integration in the nuclear genome as fusion genes equipped with targeting signals that direct the import of the protein product into organelles. With regard to the general feasibility of expressing foreign proteins in microalgae, it appears that despite the considerable body of knowledge accumulated, inducing high expression of transgenes is difficult, possibly due to epigenetic mechanisms. Recent studies have made important improvements for overcoming this problem. Expanding the use of microalga for applied and basic research will benefit from the development of better protocols for gene knock-down by RNAi, as well as for successful deletion of specific genes by gene replacement. The recent revolution in genome editing provided by the CRISPR-Cas9 system should be adapted to microalgae in the coming years. Finally, the improvement of systems for inducible expression is also of great importance, since this will allow us to control the temporal expression of toxic molecules. Finally, rapid advancements in genome sequencing, now routinely performed for all organisms, will prove instrumental in advancing these goals in the near future.

## Author contributions

LS, NS, and MS all made literature surveys and participated in the writing of the review.

## Funding

MS is incumbent of the Myles Thaler Chair in Plant Genetics. NS was supported by the Gazit-Globe fellowship.

### Conflict of interest statement

The authors declare that the review article was written in the absence of any commercial or financial relationships that could be construed as a potential conflict of interest. The reviewer, KG, and handling Editor declared their shared affiliation, and the handling Editor states that the process nevertheless met the standards of a fair and objective review.

## References

[B1] Adam Z. Rudella A. van Wijk K. J. (2006). Recent advances in the study of Clp, FtsH and other proteases located in chloroplasts. Curr. Opin. Plant Biol. 9, 234–240. 10.1016/j.pbi.2006.03.010 16603408

[B2] Afi L. Metzger P. Largeau C. Connan J. Berkaloff C. Rousseau B. (1996). Bacterial degradation of green microalgae: incubation of *Chlorella emersonii* and *Chlorella vulgaris* with *Pseudomonas oleovorans* and *Flavobacterium aquatile*. Org. Geochem. 25, 117–130. 10.1016/S0146-6380(96)00113-1

[B3] Apt K. E. Grossman A. Kroth-Pancic P. (1996). Stable nuclear transformation of the diatom *Phaeodactylum tricornutum*. Mol. Gen. Genet. 252, 572–579. 8914518 10.1007/BF02172403

[B4] Arad S. M. Levy-Ontman O. (2010). Red microalgal cell-wall polysaccharides: biotechnological aspects. Curr. Opin. Biotechnol. 21, 358–364. 10.1016/j.copbio.2010.02.008 20219344

[B5] Baek K. Lee Y. Nam O. Park S. Sim S. J. Jin E. (2016). Introducing *Dunaliella* LIP promoter containing light-inducible motifs improves transgenic expression in *Chlamydomonas reinhardtii*. Biotechnol. J. 11, 384–392. 10.1002/biot.201500269 26773277

[B6] Bai L. L. Yin W. B. Chen Y. H. Niu L. L. Sun Y. R. Zhao S. M.. (2013). A new strategy to produce a defensin: stable production of mutated NP-1 in nitrate reductase-deficient *Chlorella ellipsoidea*. PLoS ONE 8:e54966. 10.1371/journal.pone.0054966 23383016 PMC3557228

[B7] Barahimipour R. Neupert J. Bock R. (2016). Efficient expression of nuclear transgenes in the green alga *Chlamydomonas:* synthesis of an HIV antigen and development of a new selectable marker. Plant Mol. Biol. 90, 403–418. 10.1007/s11103-015-0425-8 26747175 PMC4766212

[B8] Barnes D. Franklin S. Schultz J. Henry R. Brown E. Coragliotti A.. (2005). Contribution of 5′- and 3′-untranslated regions of plastid mRNAs to the expression of *Chlamydomonas reinhardtii* chloroplast genes. Mol. Genet. Genomics 274, 625–636. 10.1007/s00438-005-0055-y 16231149

[B9] Bateman J. M. Purton S. (2000). Tools for chloroplast transformation in *Chlamydomonas*: expression vectors and a new dominant selectable marker. Mol. Gen. Genet. 263, 404–410. 10.1007/s004380051184 10821174

[B10] Becker B. Marin B. (2009). Streptophyte algae and the origin of embryophytes. Ann. Bot. 103, 999–1004. 10.1093/aob/mcp044 19273476 PMC2707909

[B11] Ben-Amotz A. Avron M. (1972). Photosynthetic activities of the halophilic alga *Dunaliella parva*. Plant Physiol. 49, 240–243. 16657932 10.1104/pp.49.2.240 PMC365936

[B12] Ben-Amotz A. Avron M. (1983). On the factors which determine massive beta-carotene accumulation in the halotolerant alga *Dunaliella bardawil*. Plant Physiol. 72, 593–597. 10.1104/pp.72.3.593 16663050 PMC1066285

[B13] Berges J. (1997). Miniview: algal nitrate reductases. Eur. J. Phycol. 32, 3–8. 10.1080/09541449710001719315

[B14] Berthold P. Schmitt R. Mages W. (2002). An engineered *Streptomyces hygroscopicus* aph 7” gene mediates dominant resistance against hygromycin B in *Chlamydomonas reinhardtii*. Protist 153, 401–412. 10.1078/14344610260450136 12627869

[B15] Bingham S. E. Cox J. C. Strem M. D. (1989). Expression of foreign DNA in *Chlamydomonas reinhardtii*. FEMS Microbiol. Lett. 53, 77–81. 10.1111/j.1574-6968.1989.tb03600.x 2558951

[B16] Blaby I. K. Blaby-Haas C. E. Tourasse N. Hom E. F. Lopez D. Aksoy M.. (2014). The *Chlamydomonas* genome project: a decade on. Trends Plant Sci. 19, 672–680. 10.1016/j.tplants.2014.05.008 24950814 PMC4185214

[B17] Blankenship J. E. Kindle K. L. (1992). Expression of chimeric genes by the light-regulated cabII-1 promoter in *Chlamydomonas reinhardtii*: a cabII-1/nit1 gene functions as a dominant selectable marker in a nit1-nit2-strain. Mol. Cell. Biol. 12, 5268–5279. 10.1128/MCB.12.11.5268 1406696 PMC360460

[B18] Bock R. (2015). Engineering plastid genomes: methods, tools, and applications in basic research and biotechnology. Annu. Rev. Plant Biol. 66, 211–241. 10.1146/annurev-arplant-050213-040212 25494465

[B19] Bocobza S. E. Aharoni A. (2014). Small molecules that interact with RNA: riboswitch based gene control and its involvement in metabolic regulation in plants and algae. Plant J. 79, 693–703. 10.1111/tpj.12540 24773387

[B20] Bocobza S. E. Malitsky S. Araujo W. L. Nunes-Nesi A. Meir S. Shapira M.. (2013). Orchestration of thiamin biosynthesis and central metabolism by combined action of the thiamin pyrophosphate riboswitch and the circadian clock in *Arabidopsis*. Plant Cell 25, 288–307. 10.1105/tpc.112.106385 23341335 PMC3584542

[B21] Bocobza S. Adato A. Mandel T. Shapira M. Nudler E. Aharoni A. (2007). Riboswitch-dependent gene regulation and its evolution in the plant kingdom. Gene Dev. 21, 2874–2879. 10.1101/gad.443907 18006684 PMC2049190

[B22] Boger P. Sandmann G. (1998). Carotenoid biosynthesis inhibitor herbicides-mode of action and resistance mechanisms. Pesticide Outlook 9, 25–39.

[B23] Borowitzka M. A. (2013). High-value products from microalgae - their development and commercialisation. J. Appl. Phycol. 25, 743–756. 10.1007/s10811-013-9983-9

[B24] Bowler C. Allen A. E. Badger J. H. Grimwood J. Jabbari K. Kuo A.. (2008). The *Phaeodactylum* genome reveals the evolutionary history of diatom genomes. Nature 456, 239–244. 10.1038/nature07410 18923393

[B25] Boynton J. Gillham N. Harris E. Hosler J. Johnson A. Jones A.. (1988). Chloroplast transformation in *Chlamydomonas* with high velocity microprojectiles. Science 240, 1534–1538. 10.1126/science.2897716 2897716

[B26] Braun E. Aach H. G. (1975). Enzymatic degradation of the cell wall of *Chlorella*. Planta 126, 181–18 *5*. 10.1007/BF00380622 24430161

[B27] Breaker R. R. (2012). Riboswitches and the RNA world. Cold Spring Harb. Perspect. Biol. 4:a003566. 10.1101/cshperspect.a003566 21106649 PMC3281570

[B28] Brown L. E. Sprecher S. Keller L. (1991). Introduction of exogenous DNA into *Chlamydomonas reinhardtii* by electroporation. Mol. Cell. Biol. 11, 2328–2332. 10.1128/MCB.11.4.2328 2005916 PMC359944

[B29] Bruggeman A. J. Kuehler D. Weeks D. P. (2014). Evaluation of three herbicide resistance genes for use in genetic transformations and for potential crop protection in algae production. Plant Biotechnol. J. 12, 894–902. 10.1111/pbi.12192 24796724

[B30] Cadoret J.-P. Garnier M. Saint-Jean B. (2012). Chapter eight - microalgae, functional genomics and biotechnology, in Advances in Botanical Research, ed Gwenaël P. (Academic Press), 285–341.

[B31] Cerutti H. Johnson A. M. Gillham N. W. Boynton J. E. (1997a). Epigenetic silencing of a foreign gene in nuclear transformants of *Chlamydomonas*. Plant Cell 9, 925–945. 10.1105/tpc.9.6.925 9212467 PMC156968

[B32] Cerutti H. Johnson A. M. Gillham N. W. Boynton J. E. (1997b). A eubacterial gene conferring spectinomycin resistance on *Chlamydomonas reinhardtii*: integration into the nuclear genome and gene expression. Genetics 145, 97. 9017393 10.1093/genetics/145.1.97 PMC1207788

[B33] Cerutti H. Ma X. Msanne J. Repas T. (2011). RNA-mediated silencing in algae: biological roles and tools for analysis of gene function. Eukaryot. Cell 10, 1164–1172. 10.1128/EC.05106-11 21803865 PMC3187060

[B34] Cha T. S. Chen C.-F. Yee W. Aziz A. Loh S.-H. (2011). Cinnamic acid, coumarin and vanillin: alternative phenolic compounds for efficient *Agrobacterium*-mediated transformation of the unicellular green alga, *Nannochloropsis* sp. J. Microbiol. Methods 84, 430–434. 10.1016/j.mimet.2011.01.005 21256888

[B35] Cha T. S. Yee W. Aziz A. (2012). Assessment of factors affecting Agrobacterium-mediated genetic transformation of the unicellular green alga, *Chlorella vulgaris*. World J. Microbiol. Biotechnol. 28, 1771–1779. 10.1007/s11274-011-0991-0 22805959

[B36] Chaumont D. (1993). Biotechnology of algal biomass production: a review of systems for outdoor mass culture. J. Appl. Phycol. 5, 593–604. 10.1007/BF02184638

[B37] Chen H. L. Li S. S. Huang R. Tsai H. J. (2008). Conditional production of a functional fish growth hormone in the transgenic line of *Nannochloropsis oculata* (Eustigmatophyceae). J. Phycol. 44, 768–776. 10.1111/j.1529-8817.2008.00508.x 27041435

[B38] Chen Y. Wang Y. Q. Sun Y. R. Zhang L. M. Li W. B. (2001). Highly efficient expression of rabbit neutrophil peptide-1 gene in *Chlorella ellipsoidea* cells. Curr. Genet. 39, 365–370. 10.1007/s002940100205 11525411

[B39] Cheney D. P. Mar E. Saga N. van der Meer J. (1986). Protoplast isolation and cell division in the agar-producing seaweed *Gracilaria (*Rhodophyta) J. Phycol. 22, 238–243. 10.1111/j.1529-8817.1986.tb04169.x

[B40] Cheng Q. Day A. Dowson-Day M. Shen G.-F. Dixon R. (2005). The *Klebsiella pneumoniae* nitrogenase Fe protein gene (nifH) functionally substitutes for the chlL gene in *Chlamydomonas reinhardtii*. Biochem. Biophys. Res. Commun. 329, 966–975. 10.1016/j.bbrc.2005.02.064 15752750

[B41] Cheng Q. Hallmann A. Edwards L. Miller S. M. (2006). Characterization of a heat-shock-inducible hsp70 gene of the green alga *Volvox carteri*. Gene 371, 112–120. 10.1016/j.gene.2005.11.026 16476527

[B42] Cheng R. Ma R. Li K. Rong H. Lin X. Wang Z. (2012). *Agrobacterium tumefaciens* mediated transformation of marine microalgae *>Schizochytrium*. Microbiol. Res. 167, 179–186. 10.1016/j.micres.2011.05.003 21641193

[B43] Chipman D. Barak Z. Schloss J. V. (1998). Biosynthesis of 2-aceto-2-hydroxy acids: acetolactate synthases and acetohydroxyacid synthases. Biochim. Biophys. Acta 1385, 401–419. 10.1016/S0167-4838(98)00083-1 9655946

[B44] Chow K.-C. Tung W. (1999). Electrotransformation of *Chlorella vulgaris*. Plant Cell Rep. 18, 778–780. 10.1007/s002990050660

[B45] Cohen E. Koren A. Arad S. (1991). A closed system for outdoor cultivation of microalgae. Biomass Bioenergy 1, 83–88. 10.1016/0961-9534(91)90030-G

[B46] Cordero B. F. Couso I. León R. Rodríguez H. Vargas M. Á. (2011). Enhancement of carotenoids biosynthesis in *Chlamydomonas reinhardtii* by nuclear transformation using a phytoene synthase gene isolated from *Chlorella zofingiensis*. Appl. Microbiol. Biotechnol. 91, 341–351. 10.1007/s00253-011-3262-y 21519934 PMC3125507

[B47] Cui Y. Qin S. Jiang P. (2014). Chloroplast transformation of *Platymonas* (*Tetraselmis*) *subcordiformis* with the *bar* gene as selectable marker. PLoS ONE 9:e98607. 10.1371/journal.pone.0098607 24911932 PMC4049664

[B48] Daniell H. Lee S. B. Panchal T. Wiebe P. O. (2001). Expression of the native cholera toxin B subunit gene and assembly as functional oligomers in transgenic tobacco chloroplasts. J. Mol. Biol. 311, 1001–1009. 10.1006/jmbi.2001.4921 11531335 PMC3473180

[B49] Dauvillée D. Delhaye S. Gruyer S. Slomianny C. Moretz S. E. d'Hulst C.. (2010). Engineering the chloroplast targeted malarial vaccine antigens in *Chlamydomonas* starch granules. PLoS ONE 5:e15424. 10.1371/journal.pone.0015424 21179538 PMC3002285

[B50] Dawson H. N. Burlingame R. Cannons A. C. (1997). Stable transformation of *Chlorella*: rescue of nitrate reductase-deficient mutants with the nitrate reductase gene. Curr. Microbiol. 35, 356–362. 10.1007/s002849900268 9353220

[B51] Day A. Debuchy R. Dillewijn J. Purton S. Rochaix J. D. (1990). Studies on the maintenance and expression of cloned DNA fragments in the nuclear genome of the green alga *Chlamydomonas reinhardtii*. Physiol. Plant. 78, 254–260. 10.1111/j.1399-3054.1990.tb02089.x

[B52] De Riso V. Raniello R. Maumus F. Rogato A. Bowler C. Falciatore A. (2009). Gene silencing in the marine diatom *Phaeodactylum tricornutum*. Nucleic Acids Res. 37, e96. 10.1093/nar/gkp448 19487243 PMC2724275

[B53] Debuchy R. Purton S. Rochaix J. (1989). The argininosuccinate lyase gene of *Chlamydomonas reinhardtii*: an important tool for nuclear transformation and for correlating the genetic and molecular maps of the ARG7 locus. EMBO J. 8, 2803. 2583083 10.1002/j.1460-2075.1989.tb08426.x PMC401327

[B54] Diaz M. Mora V. Pedrozo F. Nichela D. Baffico G. (2015). Evaluation of native acidophilic algae species as potential indicators of polycyclic aromatic hydrocarbon (PAH) soil contamination. J. Appl. Phycol. 27, 321–325. 10.1007/s10811-014-0334-2

[B55] Diaz-Santos E. de la Vega M. Vila M. Vigara J. Leon R. (2013). Efficiency of different heterologous promoters in the unicellular microalga *Chlamydomonas reinhardtii*. Biotechnol. Prog. 29, 319–328. 10.1002/btpr.1690 23319236

[B56] Doebbe A. Rupprecht J. Beckmann J. Mussgnug J. H. Hallmann A. Hankamer B.. (2007). Functional integration of the HUP1 hexose symporter gene into the genome of *C. reinhardtii*: Impacts on biological H2 production. J. Biotechnol. 131, 27–33. 10.1016/j.jbiotec.2007.05.017 17624461

[B57] Doetsch N. A. Favreau M. R. Kuscuoglu N. Thompson M. D. Hallick R. B. (2001). Chloroplast transformation in *Euglena gracilis*: splicing of a group III twintron transcribed from a transgenic psbK operon. Curr. Genet. 39, 49–60. 10.1007/s002940000174 11318107

[B58] Doran P. M. (2006). Foreign protein degradation and instability in plants and plant tissue cultures. Trends Biotechnol. 24, 426–432. 10.1016/j.tibtech.2006.06.012 16843560

[B59] Dreesen I. A. J. Hamri G. C.-E. Fussenegger M. (2010). Heat-stable oral alga-based vaccine protects mice from *Staphylococcus aureus* infection. J. Biotechnol. 145, 273–280. 10.1016/j.jbiotec.2009.12.006 19995584

[B60] Dunahay T. (1993). Transformation of *Chlamydomonas reinhardtii* with silicon carbide whiskers. Biotechniques 15, 452–455, 457–458, 460. 8217158

[B61] Dunahay T. Jarvis E. Dais S. Roessler P. (1996). Manipulation of microalgal lipid production using genetic engineering, in Seventeenth Symposium on Biotechnology for Fuels and Chemicals, eds Wyman C. Davison B. (New York, NY: Springer Science+Business Media), 223–231. 10.1007/978-1-4612-0223-3_20

[B62] Dunahay T. G. Jarvis E. E. Roessler P. G. (1995). Genetic transformation of the diatoms *Cyclotella cryptica* and *Navicula saprophila*. J. Phycol. 31, 1004–1012. 10.1111/j.0022-3646.1995.01004.x

[B63] Eberhard S. Drapier D. Wollman F. A. (2002). Searching limiting steps in the expression of chloroplast-encoded proteins: relations between gene copy number, transcription, transcript abundance and translation rate in the chloroplast of *Chlamydomonas reinhardtii*. Plant J. 31, 149–160. 10.1046/j.1365-313X.2002.01340.x 12121445

[B64] Ebnet E. Fischer M. Deininger W. Hegemann P. (1999). Volvoxrhodopsin, a light-regulated sensory photoreceptor of the spheroidal green alga *Volvox carteri*. Plant Cell 11, 1473–1484. 10.1105/tpc.11.8.1473 10449581 PMC144291

[B65] Economou C. Wannathong T. Szaub J. Purton S. (2014). A simple, low-cost method for chloroplast transformation of the green alga *Chlamydomonas reinhardtii*, in Chloroplast Biotechnology, Vol. 1132, ed Maliga P. (New York, NY: Humana Press), 401–411. 10.1007/978-1-62703-995-6_27 24599870

[B66] Eichler-Stahlberg A. Weisheit W. Ruecker O. Heitzer M. (2009). Strategies to facilitate transgene expression in Chlamydomonas reinhardtii. Planta 229, 873–883. 10.1007/s00425-008-0879-x 19127370

[B67] Elghabi Z. Ruf S. Bock R. (2011). Biolistic co-transformation of the nuclear and plastid genomes. Plant J. 67, 941–948. 10.1111/j.1365-313X.2011.04631.x 21554457

[B68] El-Sheekh M. (1999). Stable transformation of the intact cells of *Chlorella kessleri* with high velocity microprojectiles. Biol. Plant. 42, 209–216. 10.1023/A:1002104500953

[B69] Emadpour M. Karcher D. Bock R. (2015). Boosting riboswitch efficiency by RNA amplification. Nucleic Acids Res. 43,e66. 10.1093/nar/gkv165 25824954 PMC4446413

[B70] Emanuelsson O. (2002). Predicting protein subcellular localisation from amino acid sequence information. Brief. Bioinformatics 3, 361–376. 10.1093/bib/3.4.361 12511065

[B71] Emanuelsson O. Nielsen H. Brunak S. von Heijne G. (2000). Predicting subcellular localization of proteins based on their N-terminal amino acid sequence. J. Mol. Biol. 300, 1005–1016. 10.1006/jmbi.2000.3903 10891285

[B72] Emanuelsson O. Nielsen H. von Heijne G. (1999). ChloroP, a neural network-based method for predicting chloroplast transit peptides and their cleavage sites. Protein Sci. 8, 978–984. 10.1110/ps.8.5.978 10338008 PMC2144330

[B73] Fabian M. R. Sonenberg N. Filipowicz W. (2010). Regulation of mRNA translation and stability by microRNAs. Ann. Rev. Biochem. 79, 351–379. 10.1146/annurev-biochem-060308-103103 20533884

[B74] Falciatore A. Casotti R. Leblanc C. Abrescia C. Bowler C. (1999). Transformation of nonselectable reporter genes in marine diatoms. Mar. Biotechnol. 1, 239–251. 10.1007/PL00011773 10383998

[B75] Fernandez E. Schnell R. Ranum L. Hussey S. C. Silflow C. D. Lefebvre P. A. (1989). Isolation and characterization of the nitrate reductase structural gene of Chlamydomonas reinhardtii. Proc. Natl. Acad. Sci. U.S.A. 86, 6449–6453. 10.1073/pnas.86.17.6449 2475871 PMC297861

[B76] Ferrante P. Diener D. R. Rosenbaum J. L. Giuliano G. (2011). Nickel and low CO2-controlled motility in *Chlamydomonas* through complementation of a paralyzed flagella mutant with chemically regulated promoters. BMC Plant Biol. 11:22. 10.1186/1471-2229-11-22 21266063 PMC3038898

[B77] Ferris P. J. (1995). Localization of the nic-7, ac-29 and thi-10 genes within the mating-type locus of *Chlamydomonas reinhardtii*. Genetics 141, 543–549. 8647391 10.1093/genetics/141.2.543 PMC1206754

[B78] Fischer H. Robl I. Sumper M. Kröger N. (1999). Targeting and covalent modification of cell wall and membrane proteins heterologously expressed in the diatom *Cylindrotheca fusiformis* (Bacillariophyceae). J. Phycol. 35, 113–120. 10.1046/j.1529-8817.1999.3510113.x

[B79] Fischer N. Rochaix J.-D. (2001). The flanking regions of PsaD drive efficient gene expression in the nucleus of the green alga *Chlamydomonas reinhardtii*. Mol. Genet. Genomics 265, 888–894. 10.1007/s004380100485 11523806

[B80] Fischer N. Stampacchia O. Redding K. Rochaix J. D. (1996). Selectable marker recycling in the chloroplast. Mol. Gen. Genet. 251, 373–380. 10.1007/BF02172529 8676881

[B81] Franklin S. Ngo B. Efuet E. Mayfield S. P. (2002). Development of a GFP reporter gene for *Chlamydomonas reinhardtii* chloroplast. Plant J. 30, 733–744. 10.1046/j.1365-313X.2002.01319.x 12061904

[B82] Franzen L. G. Rochaix J. D. von Heijne G. (1990). Chloroplast transit peptides from the green alga *Chlamydomonas reinhardtii* share features with both mitochondrial and higher plant chloroplast presequences. FEBS Lett. 260, 165–168. 10.1016/0014-5793(90)80094-Y 2404796

[B83] Fuhrmann M. Oertel W. Hegemann P. (1999). A synthetic gene coding for the green fluorescent protein (GFP) is a versatile reporter in *Chlamydomonas reinhardtii*. Plant J. 19, 353–361. 10.1046/j.1365-313X.1999.00526.x 10476082

[B84] Gassel S. Breitenbach J. Sandmann G. (2014). Genetic engineering of the complete carotenoid pathway towards enhanced astaxanthin formation in *Xanthophyllomyces dendrorhous* starting from a high-yield mutant. Appl. Microbiol. Biotechnol. 98, 345–350. 10.1007/s00253-013-5358-z 24241897

[B85] Geng D. Wang Y. Wang P. Li W. Sun Y. (2003). Stable expression of hepatitis B surface antigen gene in *Dunaliella salina* (Chlorophyta). J. Appl. Phycol. 15, 451–456. 10.1023/B:JAPH.0000004298.89183.e5

[B86] Geng D. G. Han Y. Wang Y. Q. Wang P. Zhang L. M. Li W. B. (2004). Construction of a system for the stable expression of foreign genes in *Dunaliella salina*. Acta Bot. Sin. 46, 342–346.

[B87] Georgianna D. R. Hannon M. J. Marcuschi M. Wu S. Botsch K. Lewis A. J. (2013). Production of recombinant enzymes in the marine alga *Dunaliella tertiolecta*. Algal Res. 2, 2–9. 10.1016/j.algal.2012.10.004

[B88] Georgianna D. R. Mayfield S. P. (2012). Exploiting diversity and synthetic biology for the production of algal biofuels. Nature 488, 329–335. 10.1038/nature11479 22895338

[B89] Ghildiyal M. Zamore P. D. (2009). Small silencing RNAs: an expanding universe. Nat. Rev. Genet. 10, 94–108. 10.1038/nrg2504 19148191 PMC2724769

[B90] Goldschmidt-Clermont M. (1991). Transgenic expression of aminoglycoside adenine transferase in the chloroplast: a selectable marker of site-directed transformation of *Chlamydomonas*. Nucleic Acids Res. 19, 4083–4089. 10.1093/nar/19.15.4083 1651475 PMC328544

[B91] Goldschmidt-Clermont M. Rahire M. (1986). Sequence, evolution and differential expression of the two genes encoding variant small subunits of ribulose bisphosphate carboxylase/oxygenase in *Chlamydomonas reinhardtii*. J. Mol. Biol. 191, 421–432. 10.1016/0022-2836(86)90137-3 3820291

[B92] Gregory J. A. Li F. Tomosada L. M. Cox C. J. Topol A. B. Vinetz J. M.. (2012). Algae-produced Pfs25 elicits antibodies that inhibit malaria transmission. PLoS ONE 7:e37179. 10.1371/journal.pone.0037179 22615931 PMC3353897

[B93] Gregory J. A. Topol A. B. Doerner D. Z. Mayfield S. (2013). Alga-produced cholera toxin-Pfs25 fusion proteins as oral vaccines. Appl. Environ. Microbiol. 79, 3917–3925. 10.1128/AEM.00714-13 23603678 PMC3697563

[B94] Gruber A. Rocap G. Kroth P. G. Armbrust E. V. Mock T. (2015). Plastid proteome prediction for diatoms and other algae with secondary plastids of the red lineage. Plant J. 81, 519–528. 10.1111/tpj.12734 25438865 PMC4329603

[B95] Grundman O. Khozin-Goldberg I. Raveh D. Cohen Z. Vyazmensky M. Boussiba S.. (2012). Cloning, mutagenesis, and characterization of the microalga *Parietochloris incisa* acetohydroxyacid synthase, and its possible use as an endogenous selection marker. Biotechnol. Bioeng. 109, 2340–2348. 10.1002/bit.24515 22488216

[B96] Guo S.-L. Zhao X.-Q. Tang Y. Wan C. Alam M. A. Ho S.-H.. (2013). Establishment of an efficient genetic transformation system in *Scenedesmus obliquus*. J. Biotechnol. 163, 61–68. 10.1016/j.jbiotec.2012.10.020 23147423

[B97] Gutiérrez C. L. Gimpel J. Escobar C. Marshall S. H. Henríquez V. (2012). Chloroplast genetic tool for the green microalgae *Haematococcus pluvialis* (Chlorophyceae, Volvocales). J. Phycol. 48, 976–983. 10.1111/j.1529-8817.2012.01178.x 27009007

[B98] Habib S. J. Neupert W. Rapaport D. (2007). Analysis and prediction of mitochondrial targeting signals. Methods Cell Biol. 80, 761–781. 10.1016/S0091-679X(06)80035-X 17445721

[B99] Hagedorn M. Carter V. L. (2015). Seasonal preservation success of the marine Dinoflagellate coral symbiont, *Symbiodinium* sp. PLoS ONE 10: e0136358. 10.1371/journal.pone.0136358 26422237 PMC4589415

[B100] Hall L. M. Taylor K. B. Jones D. D. (1993). Expression of a foreign gene in *Chlamydomonas reinhardtii*. Gene 124, 75–81. 10.1016/0378-1119(93)90763-S 8382657

[B101] Hallmann A. (2007). Algal transgenics and biotechnology. Transgen. Plant J. 1, 81–98. Available online at: http://www.globalsciencebooks.info/Online/GSBOnline/images/0706/TPJ_1(1)/TPJ_1(1)81-98o.pdf

[B102] Hallmann A. Rappel A. (1999). Genetic engineering of the multicellular green alga*Volvox*: a modified and multiplied bacterial antibiotic resistance gene as a dominant selectable marker. Plant J. 17, 99–109. 10.1046/j.1365-313X.1999.00342.x 10069071

[B103] Hallmann A. Rappel A. Sumper M. (1997). Gene replacement by homologous recombination in the multicellular green alga *Volvox carteri*. Proc. Natl. Acad. Sci. U.S.A. 94, 7469–7474. 10.1073/pnas.94.14.7469 9207115 PMC23845

[B104] Hallmann A. Sumper M. (1996). The Chlorella hexose/H+ symporter is a useful selectable marker and biochemical reagent when expressed in *Volvox*. Proc. Natl. Acad. Sci. U.S.A. 93, 669–673. 10.1073/pnas.93.2.669 8570613 PMC40110

[B105] Hallmann A. Wodniok S. (2006). Swapped green algal promoters: aphVIII-based gene constructs with *Chlamydomonas* flanking sequences work as dominant selectable markers in *Volvox* and vice versa. Plant Cell Rep. 25, 582–591. 10.1007/s00299-006-0121-x 16456645

[B106] Haring M. A. Beck C. F. (1997). A promoter trap for *Chlamydomonas reinhardtii*: development of a gene cloning method using 5′ RACE based probes. Plant J. 11, 1341–1348. 10.1046/j.1365-313X.1997.11061341.x 9225472

[B107] Harris E. (2009). Chlamydomonas Sourcebook: Introduction to Chlamydomonas and Its Laboratory Use. San Diego, CA: Academic Press.

[B108] Harris E. H. Burkhart B. D. Gillham N. W. Boynton J. E. (1989). Antibiotic resistance mutations in the chloroplast 16S and 23S rRNA genes of *Chlamydomonas reinhardtii*: correlation of genetic and physical maps of the chloroplast genome. Genetics 123, 281–292. 2583478 10.1093/genetics/123.2.281 PMC1203800

[B109] Hawkins R. L. Nakamura M. (1999). Expression of human growth hormone by the eukaryotic alga, *Chlorella*. Curr. Microbiol. 38, 335–341. 10.1007/PL00006813 10341074

[B110] Hayashi Y. Shinozaki A. (2012). Visualization of microbodies in *Chlamydomonas reinhardtii*. J. Plant Res. 125, 579–586. 10.1007/s10265-011-0469-z 22205201

[B111] Heitzer M. Eckert A. Fuhrmann M. Griesbeck C. (2007). Influence of codon bias on the expression of foreign genes in microalgae, in Transgenic Microalgae as Green Cell Factories, Vol. 616, eds Rosa L. Aurora G. Emilio F. (New York, NY: Springer), 46–53. 10.1007/978-0-387-75532-8_5 18161490

[B112] Helliwell K. E. Scaife M. A. Sasso S. Araujo A. P. U. Purton S. Smith A. G. (2014). Unraveling vitamin B12-responsive gene regulation in algae. Plant Physiol. 165, 388–397. 10.1104/pp.113.234369 24627342 PMC4012597

[B113] Hou Q. Qiu S. Liu Q. Tian J. Hu Z. Ni J. (2013). Selenoprotein-transgenic *Chlamydomonas reinhardtii*. Nutrients 5:624. 10.3390/nu5030624 23443677 PMC3705309

[B114] Huang J. Liu J. Li Y. Chen F. (2008). Isolation and characterization of the phytoene desaturase gene as a potential selective marker for genetic engineering of the astaxanthin-producing green alga *Chlorella zofingiensis* (Chlorophyta). J. Phycol. 44, 684–690. 10.1111/j.1529-8817.2008.00511.x 27041426

[B115] Huang S. Taylor N. L. Whelan J. Millar A. H. (2009). Refining the definition of plant mitochondrial presequences through analysis of sorting signals, N-terminal modifications, and cleavage motifs. Plant Physiol. 150, 1272–1285. 10.1104/pp.109.137885 19474214 PMC2705053

[B116] Huesgen P. F. Schuhmann H. Adamska I. (2009). Deg/HtrA proteases as components of a network for photosystem II quality control in chloroplasts and cyanobacteria. Res. Microbiol. 160, 726–732. 10.1016/j.resmic.2009.08.005 19732828

[B117] Imam S. H. Snell W. J. (1988). The *Chlamydomonas* cell wall degrading enzyme, lysin, acts on two substrates within the framework of the wall. J. Cell Biol. 106, 2211–2221. 10.1083/jcb.106.6.2211 3384857 PMC2115132

[B118] Imamura S. Hagiwara D. Suzuki F. Kurano N. Harayama S. (2012). Genetic transformation of Pseudochoricystis ellipsoidea, an aliphatic hydrocarbon-producing green alga. J. Gen. Appl. Microbiol. 58, 1–10. 10.2323/jgam.58.1 22449745

[B119] Iseki M. Matsunaga S. Murakami A. Ohno K. Shiga K. Yoshida K.. (2002). A blue-light-activated adenylyl cyclase mediates photoavoidance in *Euglena gracilis*. Nature 415, 1047–1051. 10.1038/4151047a 11875575

[B120] Ishikawa T. Nishikawa H. Gao Y. Sawa Y. Shibata H. Yabuta Y.. (2008). The pathway via D-galacturonate/L-galactonate is significant for ascorbate biosynthesis in *Euglena gracilis* Identification and functional characterization of aldonolactonase. J. Biol. Chem. 283, 31133–31141. 10.1074/jbc.M803930200 18782759 PMC2662179

[B121] Ishikura K. Takaoka Y. Kato K. Sekine M. Yoshida K. Shinmyo A. (1999). Expression of a foreign gene in *Chlamydomonas reinhardtii* chloroplast. J. Biosci. Bioeng. 87, 307–314. 10.1016/S1389-1723(99)80037-1 16232473

[B122] Jaenicke L. Kuhne W. Spessert R. Wahle U. Waffenschmidt S. (1987). Cell-wall lytic enzymes (autolysins) of *Chlamydomonas reinhardtii* are (hydroxy)proline-specific proteases. Eur. J. Biochem. 170, 485–491. 10.1111/j.1432-1033.1987.tb13725.x 3319620

[B123] Jakobiak T. Mages W. Scharf B. Babinger P. Stark K. Schmitt R. (2004). The bacterial paromomycin resistance gene, aphH, as a dominant selectable marker in *Volvox carteri*. Protist 155, 381–393. 10.1078/1434461042650343 15648719

[B124] Jarvis E. E. Brown L. M. (1991). Transient expression of firefly luciferase in protoplasts of the green alga *Chlorella ellipsoidea*. Curr. Genet. 19, 317–321. 10.1007/BF00355062

[B125] Jarvis E. E. Dunahay T. G. Brown L. M. (1992). DNA nucleoside composition and methylation in several species of microalgae. J. Phycol. 28, 356–362. 10.1111/j.0022-3646.1992.00356.x

[B126] Jia Y. Li S. Allen G. Feng S. Xue L. (2012). A novel glyceraldehyde-3-phosphate dehydrogenase (GAPDH) promoter for expressing transgenes in the halotolerant alga *Dunaliella salina*. Curr. Microbiol. 64, 506–513. 10.1007/s00284-012-0102-y 22371187

[B127] Jia Y. Xue L. Liu H. Li J. (2009). Characterization of the glyceraldehyde-3-phosphate dehydrogenase (GAPDH) gene from the halotolerant alga *Dunaliella salina* and inhibition of its expression by RNAi. Curr. Microbiol. 58, 426–431. 10.1007/s00284-008-9333-3 19123030

[B128] Jiang W. Brueggeman A. J. Horken K. M. Plucinak T. M. Weeks D. P. (2014). Successful transient expression of Cas9 and single guide RNA genes in *Chlamydomonas reinhardtii*. Eukaryot. Cell 13, 1465–1469. 10.1128/EC.00213-14 25239977 PMC4248704

[B129] Jinek M. Chylinski K. Fonfara I. Hauer M. Doudna J. A. Charpentier E. (2012). A programmable dual-RNA-guided DNA endonuclease in adaptive bacterial immunity. Science 337, 816–821. 10.1126/science.1225829 22745249 PMC6286148

[B130] Jinkerson R. E. Jonikas M. C. (2015). Molecular techniques to interrogate and edit the *Chlamydomonas* nuclear genome. Plant J. 82, 393–412. 10.1111/tpj.12801 25704665

[B131] Jones C. S. Luong T. Hannon M. Tran M. Gregory J. A. Shen Z.. (2013). Heterologous expression of the C-terminal antigenic domain of the malaria vaccine candidate Pfs48/45 in the green algae *Chlamydomonas reinhardtii*. Appl. Microbiol. Biotechnol. 97, 1987–1995. 10.1007/s00253-012-4071-7 22592550

[B132] Kapler G. M. Coburn C. M. Beverley S. M. (1990). Stable transfection of the human parasite *Leishmania major* delineates a 30-kilobase region sufficient for extrachromosomal replication and expression. Mol. Cell. Biol. 10, 1084–1094. 10.1128/MCB.10.3.1084 2304458 PMC360971

[B133] Karas B. J. Diner R. E. Lefebvre S. C. McQuaid J. Phillips A. P. Noddings C. M.. (2015). Designer diatom episomes delivered by bacterial conjugation. Nature communications 6, 6925. 10.1038/ncomms7925 25897682 PMC4411287

[B134] Kathiresan S. Chandrashekar A. Ravishankar G. A. Sarada R. (2015). Regulation of astaxanthin and its intermediates through cloning and genetic transformation of beta-carotene ketolase in *Haematococcus pluvialis*. J. Biotechnol. 196–197, 33–41. 10.1016/j.jbiotec.2015.01.006 25612872

[B135] Kilian O. Kroth P. G. (2005). Identification and characterization of a new conserved motif within the presequence of proteins targeted into complex diatom plastids. Plant J. 41, 175–183. 10.1111/j.1365-313X.2004.02294.x 15634195

[B136] Kilian O. Benemann C. S. Niyogi K. K. Vick B. (2011). High-efficiency homologous recombination in the oil-producing alga *Nannochloropsis* Sp. Proc. Natl. Acad. Sci. U.S.A. 108, 21265–21269. 10.1073/pnas.1105861108 22123974 PMC3248512

[B137] Kim D. H. Kim Y. T. Cho J. J. Bae J. H. Hur S. B. Hwang I.. (2002). Stable integration and functional expression of flounder growth hormone gene in transformed microalga, *Chlorella ellipsoidea*. Mar. Biotechnol. (New York, NY) 4, 63–73. 10.1007/s1012601-0070-x 14961289

[B138] Kim E.-J. Cerutti H. (2009). Targeted gene silencing by RNA interference in *Chlamydomonas*. Methods Cell Biol. 93, 99–110. 10.1016/S0091-679X(08)93005-3 20409813

[B139] Kindle K. L. (1990). High-frequency nuclear transformation of *Chlamydomonas reinhardtii*. Proc. Natl. Acad. Sci. U.S.A. 87, 1228–1232. 10.1073/pnas.87.3.1228 2105499 PMC53444

[B140] Kindle K. L. Richards K. L. Stern D. B. (1991). Engineering the chloroplast genome: techniques and capabilities for chloroplast transformation in *Chlamydomonas reinhardtii*. Proc. Natl. Acad. Sci. U.S.A. 88, 1721–1725. 10.1073/pnas.88.5.1721 11607155 PMC51096

[B141] Kindle K. L. Schnell R. A. Fernandez E. Lefebvre P. A. (1989). Stable nuclear transformation of *Chlamydomonas* using the *Chlamydomonas* gene for nitrate reductase. J. Cell Biol. 109, 2589–2601. 10.1083/jcb.109.6.2589 2592399 PMC2115893

[B142] Kira N. Ohnishi K. Miyagawa-Yamaguchi A. Kadono T. Adachi M. (2015). Nuclear transformation of the diatom Phaeodactylum tricornutum using PCR-amplified DNA fragments by microparticle bombardment. Mar. Genomics 25, 49–56. 10.1016/j.margen.2015.12.004 26711090

[B143] Kishore G. M. Shah D. M. (1988). Amino acid biosynthesis inhibitors as herbicides. Annu. Rev. Biochem. 57, 627–663. 10.1146/annurev.bi.57.070188.003211 3052285

[B144] Koblenz B. Lechtreck K. F. (2005). The NIT1 promoter allows indu cible and reversible silencing of centrin in *Chlamydomonas reinhardtii*. Eukaryot. Cell 4, 1959–1962. 10.1128/EC.4.11.1959-1962.2005 16278463 PMC1287850

[B145] Kovar J. L. Zhang J. Funke R. P. Weeks D. P. (2002). Molecular analysis of the acetolactate synthase gene of *Chlamydomonas reinhardtii* and development of a genetically engineered gene as a dominant selectable marker for genetic transformation. Plant J. 29, 109–117. 10.1046/j.1365-313x.2002.01193.x 12060231

[B146] Kuchka M. R. Goldschmidt-Clermont M. van Dillewijn J. Rochaix J. D. (1989). Mutation at the *Chlamydomonas* nuclear *NAC2* locus specifically affects stability of the chloroplast *psbD* transcript encoding polypeptide D2 of PS II. Cell 58, 869–876. 10.1016/0092-8674(89)90939-2 2673536

[B147] Kumar S. V. Misquitta R. W. Reddy V. S. Rao B. J. Rajam M. V. (2004). Genetic transformation of the green alga—*Chlamydomonas reinhardtii* by *Agrobacterium tumefaciens*. Plant Sci. 166, 731–738. 10.1016/j.plantsci.2003.11.012

[B148] Lapidot M. Raveh D. Sivan A. Arad S. M. Shapira M. (1999). Note molecular analysis of AHAS gene of *Porphyridium* Sp. (Rhodophyta) and of a mutant resistant to sulfometuron methyl. J. Phycol. 35, 1233–1236. 10.1046/j.1529-8817.1999.3561233.x

[B149] Lapidot M. Raveh D. Sivan A. Arad S. M. Shapira M. (2002). Stable chloroplast transformation of the unicellular red alga *Porphyridium* Sp. Plant Physiol. 129, 7–12. 10.1104/pp.011023 12011332 PMC1540221

[B150] Lauersen K. J. Berger H. Mussgnug J. H. Kruse O. (2013a). Efficient recombinant protein production and secretion from nuclear transgenes in *Chlamydomonas reinhardtii*. J. Biotechnol. 167, 101–110. 10.1016/j.jbiotec.2012.10.010 23099045

[B151] Lauersen K. J. Huber I. Wichmann J. Baier T. Leiter A. Gaukel V.. (2015). Investigating the dynamics of recombinant protein secretion from a microalgal host. J. Biotechnol. 215, 62–71. 10.1016/j.jbiotec.2015.05.001 25975624

[B152] Lauersen K. J. Vanderveer T. L. Berger H. Kaluza I. Mussgnug J. H. Walker V. K.. (2013b). Ice recrystallization inhibition mediated by a nuclear-expressed and -secreted recombinant ice-binding protein in the microalga *Chlamydomonas reinhardtii*. Appl. Microbiol. Biotechnol. 97, 9763–9772. 10.1007/s00253-013-5226-x 24037309

[B153] Leon R. Fernandez E. (2007). Nuclear transformation of eukaryotic microalgae: historical overview, achievements and problems. Adv. Exp. Med. Biol. 616, 1–11. 10.1007/978-0-387-75532-8_1 18161486

[B154] León-Bañares R. González-Ballester D. Galván A. Fernández E. (2004). Transgenic microalgae as green cell-factories. Trends Biotechnol. 22, 45–52. 10.1016/j.tibtech.2003.11.003 14690622

[B155] Lerche K. Hallmann A. (2009). Stable nuclear transformation of Gonium pectorale. BMC Biotechnol. 9:64. 10.1186/1472-6750-9-64 19591675 PMC2720962

[B156] Lerche K. Hallmann A. (2013). Stable nuclear transformation of Eudorina elegans. BMC Biotechnol. 13:11. 10.1186/1472-6750-13-11 23402598 PMC3576287

[B157] Li F. Gao D. Hu H. (2014). High-efficiency nuclear transformation of the oleaginous marine *Nannochloropsis* species using PCR product. Biosci. Biotechnol. Biochem. 78, 812–817. 10.1080/09168451.2014.905184 25035984

[B158] Li J. Xue L. Yan H. Liu H. Liang J. (2008). Inducible EGFP expression under the control of the nitrate reductase gene promoter in transgenic *Dunaliella salina*. J. Appl. Phycol. 20, 137–145. 10.1007/s10811-007-9197-0

[B159] Li J. Xue L. Yan H. Wang L. Liu L. Lu Y.. (2007). The nitrate reductase gene-switch: a system for regulated expression in transformed cells of *Dunaliella salina*. Gene 403, 132–142. 10.1016/j.gene.2007.08.001 17890018

[B160] Li S. S. Tsai H. J. (2009). Transgenic microalgae as a non-antibiotic bactericide producer to defend against bacterial pathogen infection in the fish digestive tract. Fish Shellfish Immunol. 26, 316–325. 10.1016/j.fsi.2008.07.004 18691655

[B161] Li Z. Hayashimoto A. Murai N. (1992). A sulfonylurea herbicide resistance gene from *Arabidopsis thaliana* as a new selectable marker for production of fertile transgenic rice plants. Plant Physiol. 100, 662–668. 10.1104/pp.100.2.662 16653044 PMC1075610

[B162] Lin H. Kwan A. L. Dutcher S. K. (2010). Synthesizing and salvaging NAD: lessons learned from *Chlamydomonas reinhardtii*. PLoS Genet. 6:e1001105. 10.1371/journal.pgen.1001105 20838591 PMC2936527

[B163] Liu J. Sun Z. Gerken H. Huang J. Jiang Y. Chen F. (2014). Genetic engineering of the green alga *Chlorella zofingiensis:* a modified norflurazon-resistant phytoene desaturase gene as a dominant selectable marker. Appl. Microbiol. Biotechnol. 98, 5069–5079. 10.1007/s00253-014-5593-y 24584513

[B164] Liu W. S. Tang Y. L. Liu X. W. Fang T. C. (1984). Studies on the preparation and on the properties of sea snail enzymes. Hydrobiologia 116, 319–320. 10.1007/BF00027694

[B165] Liu X. Yu F. Rodermel S. (2010). Arabidopsis chloroplast FtsH, var2 and suppressors of var2 leaf variegation: a review. J. Integr. Plant Biol. 52, 750–761. 10.1111/j.1744-7909.2010.00980.x 20666930

[B166] Lumbreras V. Stevens D. R. Purton S. (1998). Efficient foreign gene expression in *Chlamydomonas reinhardtii* mediated by an endogenous intron. Plant J. 14, 441–447. 10.1046/j.1365-313X.1998.00145.x

[B167] Lutz K. A. Knapp J. E. Maliga P. (2001). Expression of bar in the plastid genome confers herbicide resistance. Plant Physiol. 125, 1585–1590. 10.1104/pp.125.4.1585 11299340 PMC88816

[B168] Malik J. Barry G. Kishore G. (1989). The herbicide glyphosate. Biofactors 2, 17–25. 2679650

[B169] Mandal M. Breaker R. R. (2004). Gene regulation by riboswitches. Nat. Rev. Mol. Cell Biol. 5, 451–463. 10.1038/nrm1403 15173824

[B170] Manuell A. L. Beligni M. V. Elder J. H. Siefker D. T. Tran M. Weber A.. (2007). Robust expression of a bioactive mammalian protein in *Chlamydomonas* chloroplast. Plant Biotechnol. J. 5, 402–412. 10.1111/j.1467-7652.2007.00249.x 17359495

[B171] Mayfield S. P. Franklin S. E. (2005). Expression of human antibodies in eukaryotic micro-algae. Vaccine 23, 1828–1832. 10.1016/j.vaccine.2004.11.013 15734050

[B172] Mayfield S. P. Franklin S. E. Lerner R. A. (2003). Expression and assembly of a fully active antibody in algae. Proc. Natl. Acad. Sci. U.S.A. 100, 438–442. 10.1073/pnas.0237108100 12522151 PMC141013

[B173] Mayfield S. P. Kindle K. L. (1990). Stable nuclear transformation of *Chlamydomonas reinhardtii* by using a *C. reinhardtii* gene as the selectable marker. Proc. Natl. Acad. Sci. U.S.A. 87, 2087–2091. 10.1073/pnas.87.6.2087 2179948 PMC53631

[B174] Mayfield S. P. Manuell A. L. Chen S. Wu J. Tran M. Siefker D.. (2007). *Chlamydomonas reinhardtii* chloroplasts as protein factories. Curr. Opin. Biotechnol. 18, 126–133. 10.1016/j.copbio.2007.02.001 17317144

[B175] Mayfield S. P. Schultz J. (2004). Development of a luciferase reporter gene, luxCt, for *Chlamydomonas reinhardtii* chloroplast. Plant J. 37, 449–458. 10.1046/j.1365-313X.2003.01965.x 14731263

[B176] Mazur B. J. Chui C. F. Smith J. K. (1987). Isolation and characterization of plant genes coding for acetolactate synthase, the target enzyme for two classes of herbicides. Plant Physiol. 85, 1110–1117. 10.1104/pp.85.4.1110 16665813 PMC1054403

[B177] Merchant S. Bogorad L. (1987). The Cu(II)-repressible plastidic cytochrome c. Cloning and sequence of a complementary DNA for the pre-apoprotein. J. Biol. Chem. 262, 9062–9067. 3036842

[B178] Merchant S. S. Prochnik S. E. Vallon O. Harris E. H. Karpowicz S. J. Witman G. B.. (2007). The *Chlamydomonas* genome reveals the evolution of key animal and plant functions. Science 318, 245–250. 10.1126/science.1143609 17932292 PMC2875087

[B179] Michelet L. Lefebvre-Legendre L. Burr S. E. Rochaix J.-D. Goldschmidt-Clermont M. (2011). Enhanced chloroplast transgene expression in a nuclear mutant of *Chlamydomonas*. Plant Biotechnol. J. 9, 565–574. 10.1111/j.1467-7652.2010.00564.x 20809927

[B180] Minoda A. Sakagami R. Yagisawa F. Kuroiwa T. Tanaka K. (2004). Improvement of culture conditions and evidence for nuclear transformation by homologous recombination in a red alga, *Cyanidioschyzon merolae* 10D. Plant Cell Physiol. 45, 667–671. 10.1093/pcp/pch087 15215501

[B181] Miyagawa A. Okami T. Kira N. Yamaguchi H. Ohnishi K. Adachi M. (2009). Research note: High efficiency transformation of the diatom *Phaeodactylum tricornutum* with a promoter from the diatom *Cylindrotheca fusiformis*. Phycol. Res. 57, 142–146. 10.1111/j.1440-1835.2009.00531.x

[B182] Miyahara M. Aoi M. Inoue-Kashino N. Kashino Y. Ifuku K. (2013). Highly efficient transformation of the diatom Phaeodactylum tricornutum by multi-pulse electroporation. Biosci. Biotechnol. Biochem. 77, 874–876. 10.1271/bbb.120936 23563551

[B183] Molnar A. Bassett A. Thuenemann E. Schwach F. Karkare S. Ossowski S.. (2009). Highly specific gene silencing by artificial microRNAs in the unicellular alga *Chlamydomonas reinhardtii*. Plant J. 58, 165–174. 10.1111/j.1365-313X.2008.03767.x 19054357

[B184] Monier A. Pagarete A. de Vargas C. Allen M. J. Read B. Claverie J. M.. (2009). Horizontal gene transfer of an entire metabolic pathway between a eukaryotic alga and its DNA virus. Genome Res. 19, 1441–1449. 10.1101/gr.091686.109 19451591 PMC2720186

[B185] Murray C. Sutherland P. W. Phung M. M. Lester M. T. Marshall R. K. Christeller J. T. (2002). Expression of biotin-binding proteins, avidin and streptavidin, in plant tissues using plant vacuolar targeting sequences. Transgenic Res. 11, 199–214. 10.1023/A:1015237610263 12054353

[B186] Nakamura Y. Gojobori T. Ikemura T. (2000). Codon usage tabulated from international DNA sequence databases: status for the year 2000. Nucleic Acids Res. 28, 292–292. 10.1093/nar/28.1.292 10592250 PMC102460

[B187] Nelson J. Lefebvre P. A. (1995). Targeted disruption of the *NIT8* gene in *Chlamydomonas reinhardtii*. Mol. Cell. Biol. 15, 5762–576 *9*. 10.1128/MCB.15.10.5762 7565729 PMC230828

[B188] Nelson J. Savereide P. B. Lefebvre P. A. (1994). The CRY1 gene in *Chlamydomonas reinhardtii*: structure and use as a dominant selectable marker for nuclear transformation. Mol. Cell. Biol. 14, 4011–4019. 10.1128/MCB.14.6.4011 8196640 PMC358767

[B189] Neupert J. Bock R. (2009). Designing and using synthetic RNA thermometers for temperature-controlled gene expression in bacteria. Nat. Protoc. 4, 1262–1273. 10.1038/nprot.2009.112 19680240

[B190] Neupert J. Karcher D. Bock R. (2009). Generation of Chlamydomonas strains that efficiently express nuclear transgenes. Plant J. 57, 1140–1150. 10.1111/j.1365-313X.2008.03746.x 19036032

[B191] Newman S. M. Boynton J. E. Gillham N. W. Randolph-Anderson B. L. Johnson A. M. Harris E. H. (1990). Transformation of chloroplast ribosomal RNA genes in *Chlamydomonas*: molecular and genetic characterization of integration events. Genetics 126, 875–888. 1981764 10.1093/genetics/126.4.875 PMC1204285

[B192] Newman S. M. Harris E. H. Johnson A. M. Boynton J. E. Gillham N. W. (1992). Nonrandom distribution of chloroplast recombination events in *Chlamydomonas Reinhardtii*: evidence for a hotspot and an adjacent cold region. Genetics 132, 413–429. 1358751 10.1093/genetics/132.2.413 PMC1205146

[B193] Nickelsen J. van Dillewijn J. Rahire M. Rochaix J.-D. (1994). Determinants for stability of the chloroplast psbD RNA are located within its short leader region in *Chlamydomonas reinhardtii*. EMBO J. 13, 3182. 8039511 10.1002/j.1460-2075.1994.tb06617.x PMC395210

[B194] Niu Y. F. Yang Z. K. Zhang M. H. Zhu C. C. Yang W. D. Liu J. S.. (2012). Transformation of diatom *Phaeodactylum tricornutum* by electroporation and establishment of inducible selection marker. Biotechniques 52, 1–3. 10.2144/000113881 26307256

[B195] Niu Y. Zhang M. Xie W. Li J. Gao Y. Yang W.. (2011). A new inducible expression system in a transformed green alga, *Chlorella vulgaris*. Genet. Mol. Res. 10, 3427–3434. 10.4238/2011.October.21.1 22033900

[B196] Nolla-Ardevol V. Strous M. Tegetmeyer H. E. (2015). Anaerobic digestion of the microalga Spirulina at extreme alkaline conditions: biogas production, metagenome, and metatranscriptome. Front. Microbiol. 6:597. 10.3389/fmicb.2015.00597 26157422 PMC4475827

[B197] Oey M. Ross I. L. Stephens E. Steinbeck J. Wolf J. Radzun K. A.. (2013). RNAi knock-down of LHCBM1, 2 and 3 increases photosynthetic H2 production efficiency of the green alga *Chlamydomonas reinhardtii*. PLoS ONE 8:e61375. 10.1371/journal.pone.0061375 23613840 PMC3628864

[B198] Ohresser M. Matagne R. F. Loppes R. (1997). Expression of the arylsulphatase reporter gene under the control of the nit1 promoter in *Chlamydomonas reinhardtii*. Curr. Genet. 31, 264–271. 10.1007/s002940050204 9065390

[B199] Ossowski S. Schwab R. Weigel D. (2008). Gene silencing in plants using artificial microRNAs and other small RNAs. Plant J. 53, 674–690. 10.1111/j.1365-313X.2007.03328.x 18269576

[B200] Ott K. H. Kwagh J. G. Stockton G. W. Sidorov V. Kakefuda G. (1996). Rational molecular design and genetic engineering of herbicide resistant crops by structure modeling and site-directed mutagenesis of acetohydroxyacid synthase. J. Mol. Biol. 263, 359–368. 10.1006/jmbi.1996.0580 8913312

[B201] Park S. Lee Y. Lee J. H. Jin E. (2013). Expression of the high light-inducible Dunaliella LIP promoter in *Chlamydomonas reinhardtii*. Planta 238, 1147–1156. 10.1007/s00425-013-1955-4 24043576

[B202] Patra K. P. Li F. Carter D. Gregory J. A. Baga S. Reed S. G.. (2015). Alga-produced malaria transmission-blocking vaccine candidate Pfs25 formulated with a human use-compatible potent adjuvant induces high-affinity antibodies that block *Plasmodium falciparum* infection of mosquitoes. Infect. Immun. 83, 1799–1808. 10.1128/IAI.02980-14 25690099 PMC4399074

[B203] Patron N. J. Waller R. F. (2007). Transit peptide diversity and divergence: a global analysis of plastid targeting signals. Bioessays 29, 1048–1058. 10.1002/bies.20638 17876808

[B204] Popper Z. A. Tuohy M. G. (2010). Beyond the green: understanding the evolutionary puzzle of plant and algal cell walls. Plant Physiol. 153, 373–383. 10.1104/pp.110.158055 20421458 PMC2879814

[B205] Poulsen N. Kroger N. (2005). A new molecular tool for transgenic diatoms: control of mRNA and protein biosynthesis by an inducible promoter-terminator cassette. FEBS J. 272, 3413–3423. 10.1111/j.1742-4658.2005.04760.x 15978046

[B206] Poulsen N. Chesley P. M. Kröger N. (2006). Molecular genetic manipulation of diatom *Thalassiosira pseudinana* (Bacillariophyceae). J. Phycol. 42, 1059–1065. 10.1111/j.1529-8817.2006.00269.x

[B207] Prasad B. Vadakedath N. Jeong H. J. General T. Cho M. G. Lein W. (2014). *Agrobacterium tumefaciens*-mediated genetic transformation of haptophytes (Isochrysis species). Appl. Microbiol. Biotechnol. 98, 8629–8639. 10.1007/s00253-014-5900-7 24993358

[B208] Pratheesh P. T. Vineetha M. Kurup G. M. (2014). An efficient protocol for the Agrobacterium-mediated genetic transformation of microalga *Chlamydomonas reinhardtii*. Mol. Biotechnol. 56, 507–515. 10.1007/s12033-013-9720-2 24198218

[B209] Priyadarshani I. Rath B. (2012). Commercial and industrial applications of micro algae–A review. J. Algal Biomass Utln. 3, 89–100. Available online at: http://jalgalbiomass.com/paper14vol3no4.pdf

[B210] Prochnik S. E. Umen J. Nedelcu A. M. Hallmann A. Miller S. M. Nishii I.. (2010). Genomic analysis of organismal complexity in the multicellular green alga *Volvox carteri*. Science 329, 223–226. 10.1126/science.1188800 20616280 PMC2993248

[B211] Przibilla E. Heiss S. Johanningmeier U. Trebst A. (1991). Site-specific mutagenesis of the D1 subunit of photosystem II in wild-type *Chlamydomonas*. Plant Cell 3, 169–174. 10.1105/tpc.3.2.169 1840907 PMC159989

[B212] Purton S. (2007). Tools and techniques for chloroplast transformation of *Chlamydomonas*. Adv. Exp. Med. Biol. 616, 34–45. 10.1007/978-0-387-75532-8_4 18161489

[B213] Purton S. Szaub J. Wannathong T. Young R. Economou C. (2013). Genetic engineering of algal chloroplasts: progress and prospects. Russ. J. Plant Physiol. 60, 491–499. 10.1134/S1021443713040146

[B214] Qin S. Sun G.-Q. Jiang P. Zou L.-H. Wu Y. Tseng C.-K. (1999). Review of genetic engineering of *Laminaria japonica* (Laminariales, Phaeophyta) in China, in Proceedings of the Sixteenth International Seaweed Symposium, Vol. 137, Series in Developments in Hydrobiology, eds Kain J. M. Brown M. T. Lahaye M. (New York, NY: Springer), 469–472. 10.1007/978-94-011-4449-0_56

[B215] Quinn J. M. Eriksson M. Moseley J. L. Merchant S. (2002). Oxygen deficiency responsive gene expression *in Chlamydomonas reinhardtii* through a copper-sensing signal transduction pathway. Plant Physiol. 128, 463–471. 10.1104/pp.010694 11842150 PMC148909

[B216] Quinn J. M. Kropat J. Merchant S. (2003). Copper response element and Crr1-dependent Ni2+-responsive promoter for induced, reversible gene expression in *Chlamydomonas reinhardtii*. Eukaryot. Cell 2, 995–1002. 10.1128/EC.2.5.995-1002.2003 14555481 PMC219375

[B217] Quinn J. M. Merchant S. (1995). Two copper-responsive elements associated with the *Chlamydomonas* Cyc6 gene function as targets for transcriptional activators. Plant Cell 7, 623–628. 10.1105/tpc.7.5.623 7780310 PMC160809

[B218] Ramundo S. Casero D. Muhlhaus T. Hemme D. Sommer F. Crevecoeur M.. (2014). Conditional depletion of the Chlamydomonas chloroplast ClpP protease activates nuclear genes involved in autophagy and plastid protein quality control. Plant Cell 26, 2201–2222. 10.1105/tpc.114.124842 24879428 PMC4079378

[B219] Ramundo S. Rahire M. Schaad O. Rochaix J.-D. (2013). Repression of essential chloroplast genes reveals new signaling pathways and regulatory feedback loops in *Chlamydomonas*. Plant Cell 25, 167–186. 10.1105/tpc.112.103051 23292734 PMC3584532

[B220] Rasala B. A. Barrera D. J. Ng J. Plucinak T. M. Rosenberg J. N. Weeks D. P.. (2013). Expanding the spectral palette of fluorescent proteins for the green microalga *Chlamydomonas reinhardtii*. Plant J. 74, 545–556. 10.1111/tpj.12165 23521393

[B221] Rasala B. A. Chao S. S. Pier M. Barrera D. J. Mayfield S. P. (2014). Enhanced genetic tools for engineering multigene traits into green algae. PLoS ONE 9:e94028. 10.1371/journal.pone.0094028 24710110 PMC3978050

[B222] Rasala B. A. Lee P. A. Shen Z. Briggs S. P. Mendez M. Mayfield S. P. (2012). Robust expression and secretion of Xylanase1 in *Chlamydomonas reinhardtii* by fusion to a selection gene and processing with the FMDV 2A peptide. PLoS ONE 7:e43349. 10.1371/journal.pone.0043349 22937037 PMC3427385

[B223] Rasala B. A. Mayfield S. P. (2014). Photosynthetic biomanufacturing in green algae; production of recombinant proteins for industrial, nutritional, and medical uses. Photosyn. Res. 123, 227–239. 10.1007/s11120-014-9994-7 24659086

[B224] Rasala B. A. Muto M. Lee P. A. Jager M. Cardoso R. M. F. Behnke C. A.. (2010). Production of therapeutic proteins in algae, analysis of expression of seven human proteins in the chloroplast of *Chlamydomonas reinhardtii*. Plant Biotechnol. J. 8, 719–733. 10.1111/j.1467-7652.2010.00503.x 20230484 PMC2918638

[B225] Reddy C. Gupta V. Jha B. (2010). Developments in biotechnology of red algae, in Red Algae in the Genomic Age, eds Seckbach J. Chapman D. J. (Dordrecht: Springer), 307–341.

[B226] Remacle C. Cline S. Boutaffala L. Gabilly S. Larosa V. Barbieri M. R.. (2009). The ARG9 gene encodes the plastid-resident N-acetyl ornithine aminotransferase in the green alga *Chlamydomonas reinhardtii*. Eukaryot. Cell 8, 1460–1463. 10.1128/EC.00108-09 19617392 PMC2747819

[B227] Richmond A. Boussiba S. Vonshak A. Kopel R. (1993). A new tubular reactor for mass production of microalgae outdoors. J. Appl. Phycol. 5, 327–332. 10.1007/BF02186235

[B228] Rochaix J.-D. Surzycki R. Ramundo S. (2014). Tools for regulated gene expression in the chloroplast of *Chlamydomonas*, in Chloroplast Biotechnology, Vol. 1132, ed Maliga P. (Humana Press), 413–424. 10.1007/978-1-62703-995-6_28 24599871

[B229] Rohr J. Sarkar N. Balenger S. Jeong B. R. Cerutti H. (2004). Tandem inverted repeat system for selection of effective transgenic RNAi strains in *Chlamydomonas*. Plant J. 40, 611–621. 10.1111/j.1365-313X.2004.02227.x 15500475

[B230] Rosenwasser S. Mausz M. A. Schatz D. Sheyn U. Malitsky S. Aharoni A.. (2014). Rewiring host lipid metabolism by large viruses determines the fate of *Emiliania huxleyi*, a bloom-forming Alga in the Ocean. Plant Cell 26, 2689–2707. 10.1105/tpc.114.125641 24920329 PMC4114960

[B231] Ryan M. D. King A. Thomas G. P. (1991). Cleavage of foot-and-mouth disease virus polyprotein is mediated by residues located within a 19 amino acid sequence. J. Gen. Virol. 72, 2727–2732. 10.1099/0022-1317-72-11-2727 1658199

[B232] Sakaue K. Harada H. Matsuda Y. (2008). Development of gene expression system in a marine diatom using viral promoters of a wide variety of origin. Physiol. Plant 133, 59–67. 10.1111/j.1399-3054.2008.01089.x 18346072

[B233] Sanger M. Daubert S. Goodman R. M. (1990). Characteristics of a strong promoter from figwort mosaic virus: comparison with the analogous 35S promoter from cauliflower mosaic virus and the regulated mannopine synthase promoter. Plant Mol. Biol. 14, 433–443. 10.1007/BF00028779 2102823

[B234] Schiedlmeier B. Schmitt R. Muller W. Kirk M. M. Gruber H. Mages W.. (1994). Nuclear transformation of *Volvox carteri*. Proc. Natl. Acad. Sci. USA. 91, 5080–5084. 10.1073/pnas.91.11.5080 8197189 PMC43935

[B235] Schmollinger S. Strenkert D. Schroda M. (2010). An inducible artificial microRNA system for *Chlamydomonas reinhardtii* confirms a key role for heat shock factor 1 in regulating thermotolerance. Curr. Genet. 56, 383–389. 10.1007/s00294-010-0304-4 20449593

[B236] Schneider G. Fechner U. (2004). Advances in the prediction of protein targeting signals. Proteomics 4, 1571–1580. 10.1002/pmic.200300786 15174127

[B237] Schroda M. (2006). RNA silencing in *Chlamydomonas*: mechanisms and tools. Curr. Genet. 49, 69–84. 10.1007/s00294-005-0042-1 16308700

[B238] Schroda M. Beck C. F. Vallon O. (2002). Sequence elements within an HSP70 promoter counteract transcriptional transgene silencing in *Chlamydomonas*. Plant J. 31, 445–455. 10.1046/j.1365-313X.2002.01371.x 12182703

[B239] Schroda M. Blöcker D. Beck C. F. (2000). The HSP70A promoter as a tool for the improved expression of transgenes in *Chlamydomonas*. Plant J. 21, 121–131. 10.1046/j.1365-313x.2000.00652.x 10743653

[B240] Sharon-Gojman R. Maimon E. Leu S. Zarka A. Boussiba S. (2015). Advanced methods for genetic engineering of *Haematococcus pluvialis* (Chlorophyceae, Volvocales). Algal Res. 10, 8–15. 10.1016/j.algal.2015.03.022

[B241] Shimogawara K. Fujiwara S. Grossman A. Usuda H. (1998). High-efficiency transformation of *Chlamydomonas reinhardtii* by electroporation. Genetics 148, 1821–1828. 9560396 10.1093/genetics/148.4.1821 PMC1460073

[B242] Sineshchekov O. A. Jung K.-H. Spudich J. L. (2002). Two rhodopsins mediate phototaxis to low-and high-intensity light in *Chlamydomonas reinhardtii*. Proc. Natl. Acad. Sci. U.S.A. 99, 8689–8694. 10.1073/pnas.122243399 12060707 PMC124360

[B243] Siripornadulsil S. Dabrowski K. Sayre R. (2007). Microalgal vaccines. Adv. Exp. Med. Biol. 616, 122–128. 10.1007/978-0-387-75532-8_11 18161496

[B244] Sizova I. Fuhrmann M. Hegemann P. (2001). A Streptomyces rimosusaphVIII gene coding for a new type phosphotransferase provides stable antibiotic resistance to *Chlamydomonas reinhardtii*. Gene 277, 221–229. 10.1016/S0378-1119(01)00616-3 11602359

[B245] Sizova I. Greiner A. Awasthi M. Kateriya S. Hegemann P. (2013). Nuclear gene targeting in *Chlamydomonas* using engineered zinc-finger nucleases. Plant J. 73, 873–882. 10.1111/tpj.12066 23137232

[B246] Small I. Peeters N. Legeai F. Lurin C. (2004). Predotar: a tool for rapidly screening proteomes for N-terminal targeting sequences. Proteomics 4, 1581–1590. 10.1002/pmic.200300776 15174128

[B247] Sodeinde O. A. Kindle K. L. (1993). Homologous recombination in the nuclear genome of *Chlamydomonas reinhardtii*. Proc. Natl. Acad. Sci. U.S.A. 90, 9199–9203. 10.1073/pnas.90.19.9199 8415677 PMC47530

[B248] Sontheimer E. J. Carthew R. W. (2005). Silence from within: endogenous siRNAs and miRNAs. Cell 122, 9–12. 10.1016/j.cell.2005.06.030 16009127

[B249] Soupene E. Inwood W. Kustu S. (2004). Lack of the Rhesus protein Rh1 impairs growth of the green alga *Chlamydomonas reinhardtii* at high CO2. Proc. Natl. Acad. Sci. U.S.A. 101, 7787–7792. 10.1073/pnas.0401809101 15096599 PMC419684

[B250] Stanne T. Sjogren L. Koussevitzky S. Clarke A. (2009). Identification of new protein substrates for the chloroplast ATP-dependent Clp protease supports its constitutive role in *Arabidopsis*. Biochem. J. 417, 257–268. 10.1042/BJ20081146 18754756

[B251] Steinbrenner J. Sandmann G. (2006). Transformation of the green alga *Haematococcus pluvialis* with a phytoene desaturase for accelerated astaxanthin biosynthesis. Appl. Environ. Microbiol. 72, 7477–7484. 10.1128/AEM.01461-06 17012596 PMC1694260

[B252] Stevens D. R. Purton S. Rochaix J.-D. (1996). The bacterial phleomycin resistance geneble as a dominant selectable marker in *Chlamydomonas*. Mol. Gen. Genet. 251, 23–30. 8628243 10.1007/BF02174340

[B253] Sugiyama M. Thompson C. J. Kumagai T. Suzuki K. Deblaere R. Villarroel R.. (1994). Characterisation by molecular cloning of two genes from *Streptomyces verticillus* encoding resistance to bleomycin. Gene 151, 11–16. 10.1016/0378-1119(94)90626-2 7530224

[B254] Sun G. Zhang X. Sui Z. Mao Y. (2008). Inhibition of pds gene expression via the RNA interference approach in *Dunaliella salina* (Chlorophyta). Mar. Biotechnol. 10, 219–226. 10.1007/s10126-007-9056-7 18204953

[B255] Sun M. Qian K. Su N. Chang H. Liu J. Shen G. (2003). Foot-and-mouth disease virus VP1 protein fused with cholera toxin B subunit expressed in *Chlamydomonas reinhardtii* chloroplast. Biotechnol. Lett. 25, 1087–1092. 10.1023/A:1024140114505 12889819

[B256] Sun X. Ouyang M. Guo J. Ma J. Lu C. Adam Z.. (2010). The thylakoid protease Deg1 is involved in photosystem-II assembly in *Arabidopsis thaliana*. Plant J. 62, 240–249. 10.1111/j.1365-313X.2010.04140.x 20088900

[B257] Sun Y. Gao X. Li Q. Zhang Q. Xu Z. (2006). Functional complementation of a nitrate reductase defective mutant of a green alga *Dunaliella viridis* by introducing the nitrate reductase gene. Gene 377, 140–149. 10.1016/j.gene.2006.03.018 16797881

[B258] Sun Y. Yang Z. Gao X. Li Q. Zhang Q. Xu Z. (2005). Expression of foreign genes in Dunaliella by electroporation. Mol. Biotechnol. 30, 185–192. 10.1385/MB:30:3:185 15988044

[B259] Surzycki R. Cournac L. Peltier G. Rochaix J. D. (2007). Potential for hydrogen production with inducible chloroplast gene expression in *Chlamydomonas*. Proc. Natl. Acad. Sci. U.S.A. 104, 17548–17553. 10.1073/pnas.0704205104 17951433 PMC2077293

[B260] Surzycki R. Greenham K. Kitayama K. Dibal F. Wagner R. Rochaix J. D.. (2009). Factors effecting expression of vaccines in microalgae. Biologicals 37, 133–138. 10.1016/j.biologicals.2009.02.005 19467445

[B261] Svab Z. Maliga P. (1993). High-frequency plastid transformation in tobacco by selection for a chimeric aadA gene. Proc. Natl. Acad. Sci. U.S.A. 90, 913–917. 10.1073/pnas.90.3.913 8381537 PMC45780

[B262] Takahashi F. Yamagata D. Ishikawa M. Fukamatsu Y. Ogura Y. Kasahara M.. (2007). Aureochrome, a photoreceptor required for photomorphogenesis in stramenopiles. Proc. Natl. Acad. Sci. U.S.A. 104, 19625–19630. 10.1073/pnas.0707692104 18003911 PMC2148339

[B263] Takeda H. (1991). Sugar composition of the cell wall and the taxonomy of *Chlorella* (Chlorophyceae) 1. J. Phycol. 27, 224–232. 10.1111/j.0022-3646.1991.00224.x

[B264] Tan C. Qin S. Zhang Q. Jiang P. Zhao F. (2005). Establishment of a micro-particle bombardment transformation system for *Dunaliella salina*. J. Microbiol. 43:361. Available online at: https://www.researchgate.net/profile/Peng_Jiang17/publication/7614955_Establishment_of_a_micro-particle_bombardment_transformation_system_for_Dunaliella_salina._J_Microbiol/links/53fcd1820cf2364ccc04dbf3.pdf 16145551

[B265] Tardif M. Atteia A. Specht M. Cogne G. Rolland N. Brugiere S.. (2012). PredAlgo: a new subcellular localization prediction tool dedicated to green algae. Mol. Biol. Evol. 29, 3625–3639. 10.1093/molbev/mss178 22826458

[B266] ten Lohuis M. R. Miller D. J. (1998). Genetic transformation of dinoflagellates (Amphidinium and Symbiodinium): expression of GUS in microalgae using heterologous promoter constructs. Plant J. 13, 427–435. 10.1046/j.1365-313X.1998.00040.x

[B267] Townsend J. A. Wright D. A. Winfrey R. J. Fu F. Maeder M. L. Joung J. K.. (2009). High-frequency modification of plant genes using engineered zinc-finger nucleases. Nature 459, 442–445. 10.1038/nature07845 19404258 PMC2743854

[B268] Tran M. Zhou B. Pettersson P. L. Gonzalez M. J. Mayfield S. P. (2009). Synthesis and assembly of a full-length human monoclonal antibody in algal chloroplasts. Biotechnol. Bioeng. 104, 663–673. 10.1002/bit.22446 19562731

[B269] Varela J. C. Pereira H. Vila M. Leon R. (2015). Production of carotenoids by microalgae: achievements and challenges. Photosyn. Res. 125, 423–436. 10.1007/s11120-015-0149-2 25921207

[B270] Viji S. Anbazhagi M. Ponpandian N. Mangalaraj D. Jeyanthi S. Santhanam P.. (2014). Diatom-based label-free optical biosensor for biomolecules. Appl. Biochem. Biotechnol. 174, 1166–1173. 10.1007/s12010-014-1040-x 24989453

[B271] Voinnet O. (2009). Origin, biogenesis, and activity of plant microRNAs. Cell 136, 669–687. 10.1016/j.cell.2009.01.046 19239888

[B272] von der Heyde E. L. Klein B. Abram L. Hallmann A. (2015). The inducible nitA promoter provides a powerful molecular switch for transgene expression in *Volvox carteri*. BMC Biotechnol. 15:5. 10.1186/s12896-015-0122-3 25888095 PMC4339647

[B273] Wachter A. Tunc-Ozdemir M. Grove B. C. Green P. J. Shintani D. K. Breaker R. R. (2007). Riboswitch control of gene expression in plants by splicing and alternative 3′ end processing of mRNAs. Plant Cell 19, 3437–3450. 10.1105/tpc.107.053645 17993623 PMC2174889

[B274] Walker T. L. Becker D. K. Collet C. (2005a). Characterisation of the *Dunaliella tertiolecta* RbcS genes and their promoter activity in *Chlamydomonas reinhardtii*. Plant Cell Rep. 23, 727–735. 10.1007/s00299-004-0884-x 15480684

[B275] Walker T. L. Becker D. K. Dale J. L. Collet C. (2005b). Towards the development of a nuclear transformation system for *Dunaliella tertiolecta*. J. Appl. Phycol. 17, 363–368. 10.1007/s10811-005-4783-5

[B276] Wang C. Wang Y. Su Q. Gao X. (2007). Transient expression of the GUS gene in a unicellular marine green alga, *Chlorella* sp. MACC/C95, via electroporation. Biotechnol. Bioprocess. Eng. 12, 180–183. 10.1007/BF03028646

[B277] Wang P. Sun Y. Li X. Zhang L. Li W. Wang Y. (2004). Rapid isolation and functional analysis of promoter sequences of the nitrate reductase gene from *Chlorella ellipsoidea*. J. Appl. Phycol. 16, 11–16. 10.1023/B:JAPH.0000019048.56489.3c

[B278] Wang T. Y. Xue L. X. Hou W. H. Yang B. S. Chai Y. R. Ji X. A.. (2007). Increased expression of transgene in stably transformed cells of *Dunaliella salina* by matrix attachment regions. Appl. Microbiol. Biotechnol. 76, 651–657. 10.1007/s00253-007-1040-7 17611755

[B279] Wang X. Brandsma M. Tremblay R. Maxwell D. Jevnikar A. Huner N.. (2008). A novel expression platform for the production of diabetes-associated autoantigen human glutamic acid decarboxylase (hGAD65). BMC Biotechnol. 8:87. 10.1186/1472-6750-8-87 19014643 PMC2621204

[B280] Wijffels R. H. (2015). The need and risks of using transgenic microalgae for the production of food, feed, chemicals and fuels, in OECD Biosafety and the Environmental Uses of Micro-Organisms: Conference Proceedings (Paris: OECD Publishing).

[B281] Xie W.-H. Zhu C.-C. Zhang N.-S. Li D.-W. Yang W.-D. Liu J.-S.. (2014). Construction of novel chloroplast expression vector and development of an efficient transformation system for the diatom *Phaeodactylum tricornutum*. Mar. Biotechnol. 16, 538–546. 10.1007/s10126-014-9570-3 24763817 PMC4169106

[B282] Yamada T. Sakaguchi K. (1982). Comparative studies on Chlorella cell walls: Induction of protoplast formation. Arch. Microbiol. 132, 10–13. 10.1007/BF00690809

[B283] Yamano T. Iguchi H. Fukuzawa H. (2013). Rapid transformation of *Chlamydomonas reinhardtii* without cell-wall removal. J. Biosci. Bioeng. 115, 691–694. 10.1016/j.jbiosc.2012.12.020 23333644

[B284] Zaslavskaia L. A. Lippmeier J. C. Kroth P. G. Grossman A. R. Apt K. E. (2000). Transformation of the diatom *Phaeodactylum tricornutum* (Bacillariophyceae) with a variety of selectable marker and reporter genes. J. Phycol. 36, 379–386. 10.1046/j.1529-8817.2000.99164.x

[B285] Zaslavskaia L. A. Lippmeier J. C. Shih C. Ehrhardt D. Grossman A. R. Apt K. E. (2001). Trophic Conversion of an obligate photoautotrophic organism through metabolic engineering. Science 292, 2073–2075. 10.1126/science.160015 11408656

[B286] Zhang C. Hu H. (2014). High-efficiency nuclear transformation of the diatom Phaeodactylum tricornutum by electroporation. Marine genomics 16, 63–66. 10.1016/j.margen.2013.10.003 24269346

[B287] Zhang R. Patena W. Armbruster U. Gang S. S. Blum S. R. Jonikas M. C. (2014). High-throughput genotyping of green algal mutants reveals random distribution of mutagenic insertion sites and endonucleolytic cleavage of transforming DNA. Plant Cell 26, 1398–1409. 10.1105/tpc.114.124099 24706510 PMC4036561

[B288] Zhao T. Wang W. Bai X. Qi Y. (2009). Gene silencing by artificial microRNAs in *Chlamydomonas*. Plant J. 58, 157–164. 10.1111/j.1365-313X.2008.03758.x 19054364

[B289] Zheng B. MacDonald T. M. Sutinen S. Hurry V. Clarke A. K. (2006). A nuclear-encoded ClpP subunit of the chloroplast ATP-dependent Clp protease is essential for early development in *Arabidopsis thaliana*. Planta 224, 1103–1115. 10.1007/s00425-006-0292-2 16705403

[B290] Zorin B. Grundman O. Khozin-Goldberg I. Leu S. Shapira M. Kaye Y. (2014). Development of a nuclear transformation system for oleaginous green alga *Lobosphaera (Parietochloris) incisa* and genetic complementation of a mutant strain, deficient in arachidonic acid biosynthesis. Plant J. 58, 157–164. 10.1371/journal.pone.0105223 PMC4136796 25133787

[B291] Zorin B. Hegemann P. Sizova I. (2005). Nuclear-gene targeting by using single-stranded DNA avoids illegitimate DNA integration in *Chlamydomonas reinhardtii*. Eukaryot. Cell 4, 1264–1272. 10.1128/EC.4.7.1264-1272.2005 16002652 PMC1168964

